# Identification and Characterization of Fully Human FOLR1-Targeting CAR T Cells for the Treatment of Ovarian Cancer

**DOI:** 10.3390/cells13221880

**Published:** 2024-11-14

**Authors:** Maria Bethke, Pierre Abramowski, Miriam Droste, André Felsberger, Lisa Kochsiek, Bettina Kotter, Luisa Plettig, Kateryna Antonova, Salpy Baghdo, Nico Burzan, Florian Tomszak, Manuel Martinez-Osuna, Dominik Eckardt, Christoph Herbel

**Affiliations:** Miltenyi Biotec B.V. & Co. KG, 51429 Bergisch Gladbach, Germany

**Keywords:** CAR T cells, FOLR1, tissue cross-reactivity, immunotherapy, spatial multiplex, lead selection, phage panning, solid cancers, immune cell engineering, high-throughput screening

## Abstract

CAR T cell therapy has been an effective treatment option for hematological malignancies. However, the therapeutic potential of CAR T cells can be reduced by several constraints, partly due to immunogenicity and toxicities. The lack of established workflows enabling thorough evaluation of new candidates, limits comprehensive CAR assessment. To improve the selection of lead CAR candidates, we established a stringent, multistep workflow based on specificity assessments, employing multiple assays and technologies. Moreover, we characterized a human FOLR1-directed CAR binding domain. Selection of binding domains was based on extensive specificity assessment by flow cytometry and imaging, to determine on-/off-target and off-tumor reactivity. CAR T cell functionality and specificity were assessed by high-throughput screening and advanced in vitro assays. Our validation strategy highlights that assays comprehensively characterizing CAR functionality and binding specificity complement each other. Thereby, critical specificity considerations can be addressed early in the development process to overcome current limitations for future CAR T cell therapies.

## 1. Introduction

The immune system protects an organism from diseases of various pathogens, e.g., viruses, bacteria, and fungi [1]. This protection depends on the ability to discriminate between the host (self) and external stimuli (non-self), as well as on directing a response to external stimuli to protect the body from harmful pathogens [2,3]. Innate immunity provides a rapid, non-specific defense by recognizing general molecular patterns on pathogens through pattern recognition receptors, which detect common structures such as bacterial cell wall components, triggering an immediate immune response. Adaptive immunity, on the other hand, offers a more targeted and specific defense by recognizing unique antigens via T and B cells, which develop memory for future encounters. Together, innate and adaptive immunity ensure effective detection and elimination of foreign invaders. Empowering the immune system to clear conditions which could not be resolved, such as cancer or autoimmune diseases, has gained attention. Coming from this idea, cellular immunotherapy and, in particular, chimeric antigen receptor (CAR)-expressing T cell therapy has provided meaningful clinical responses in many patients by redirecting T cells to target cancer cells and other pathogenic cells, such as HIV-specific CAR T cells [4,5,6].

CAR T cells are genetically modified to express an artificial receptor which combines different aspects of the physiological T cell receptor functions with a defined extracellular antigen binding domain. The CAR is modularly designed with at least one element mediating specificity towards the target antigen. Commonly, this is a single-chain variable fragment (scFv) derived from an antibody. A hinge connects the binding domain with the transmembrane part, as well as the intracellular components of the CAR, i.e., an activation domain, which is often CD3zeta, and at least one co-stimulatory domain, e.g., based on 4-1BB or CD28 [7,8].

When expressed by T cells, functional CAR constructs endow these immune cells with specific effector functions upon antigen encounter. Several seminal hallmarks of functional CAR T cells have been proposed [9,10]. After, specific CAR-dependent target recognition cytokines are produced, e.g., IFN-γ and IL-2, supporting cytotoxicity as well as CAR T cell expansion. However, the clinical cancer setting is considered more complex. It was proposed that reinvigorated T cell effector function, upon repeated antigen exposure, can serve as an in vitro surrogate to mimic the challenging patient situation [11,12,13].

Besides the promises and the impressive clinical data of CAR T cell therapy, limitations exist, which need to be overcome to further improve cellular immunotherapy for patients [14,15,16]. For instance, anti-tumor function, as well as persistence of CAR T cells, can be impacted by an anti-CAR immune reaction by the host [17,18]. Cellular and/or humoral immunogenicity of the CAR may be related to non-human protein sequences included in the CAR. Especially the use of scFv sequences, which are often derived from murine monoclonal antibodies, may pose the risk of a human anti-mouse immune reaction [19]. Recent reports describe the presence of anti-CAR antibodies and anti-CAR TCRs in some patients treated with CAR T cells, particularly when CAR T cells were applied multiple times during treatment or when different CAR products were administered in sequential treatment lines [20,21].

Another limitation of CAR T cell therapy is related to the target antigen expressed on non-malignant cells. Although extensive preclinical characterization of CAR T cells is performed, unexpected toxicities were reported for several CAR constructs [22]. For example, anti-carbonic anhydrase IX (CAIX) CAR T cells caused liver toxicities in patients with metastatic renal cell carcinoma [23,24]. Patients with carcinoembryonic antigen-related cell adhesion molecule 5 (CEACAM5)-positive solid tumors suffered from serious adverse events such as gastrointestinal, but also pulmonary, toxicity after infusion of anti-CEACAM5 CAR T cell treatment [25]. Patients receiving anti-claudin 18.2 (CLDN18.2) CAR T cells developed mucosal toxicity [26]. Epidermal growth factor receptor (EGFR)-directed CAR T cells were reported to cause mucosal and cutaneous adverse events [27,28]. Finally, patients treated with receptor tyrosine-protein kinase erbB-2 (ERBB2 or HER2)-targeting CAR T cells experienced cardiopulmonary toxicity [29].

To mitigate the risk of such toxicities, tissue cross-reactivity studies are usually performed [30,31,32]. These screening assays routinely utilize immunohistochemistry to identify non-specific and specific binding of antibodies or antibody-like proteins, in different types of human or animal tissues, respectively. Unspecific binding occurs between molecules that are not intended to interact, which can mislead decision making. One approach is the use of negative controls to establish the baseline binding level in the absence of the specific target. For instance, in antibody-based assays, including a control without the specific antigen helps identify whether the antibody binds non-specifically to other components in the sample. In immunofluorescence, elevated background fluorescence can be indicative for unspecific binding, which can be assessed using isotype controls. Additionally, in IHC, employing secondary antibody only controls can help confirm the specificity of binding. Noteworthy, the staining profiles, which are indicative for protein expression, of animal and human tissues may differ in intensity and distribution, e.g., angiotensin-converting enzyme 2, ACE2, the receptor for the severe acute respiratory syndrome coronavirus 2 (SARS-CoV-2), is expressed in human cardiomyocytes and not in murine cardiomyocytes [33]. Tissue cross-reactivity studies support the preclinical safety assessment data package.

Although conventional immunohistochemistry is widely used for tissue cross-reactivity studies, this method has some constraints, including high inter-observer variability and the use of only one single marker per tissue section [34]. In contrast, immunofluorescence (IF) offers higher sensitivity and allows for multicolor labeling, enabling the simultaneous detection of multiple proteins within a single sample. Additionally, IF provides better spatial resolution and less background staining, making it easier to visualize and quantify specific proteins in complex tissues or cells. Particularly, multiplexing and semi-automated analysis approaches can help to overcome the limitation of testing a single marker per tissue section. This improvement is of particular interest for the analysis of novel antibody fragments since the analysis of the binders and additional reference antibodies to characterize the cellular composition of the tissue can be performed on the same tissue slice. Additionally, multiplexing enables data generation from samples with limited size or scarce availability.

As an additional approach, to identify off-tumor and/or off-target toxicities in vivo, experiments in mice are conducted. However, many CAR T cells are designed with antigen-binding domains selectively recognizing human epitopes, but not murine antigens, therefore, providing only limited information on potential off-tumor targeting of these CAR T cells.

**Figure 1:**
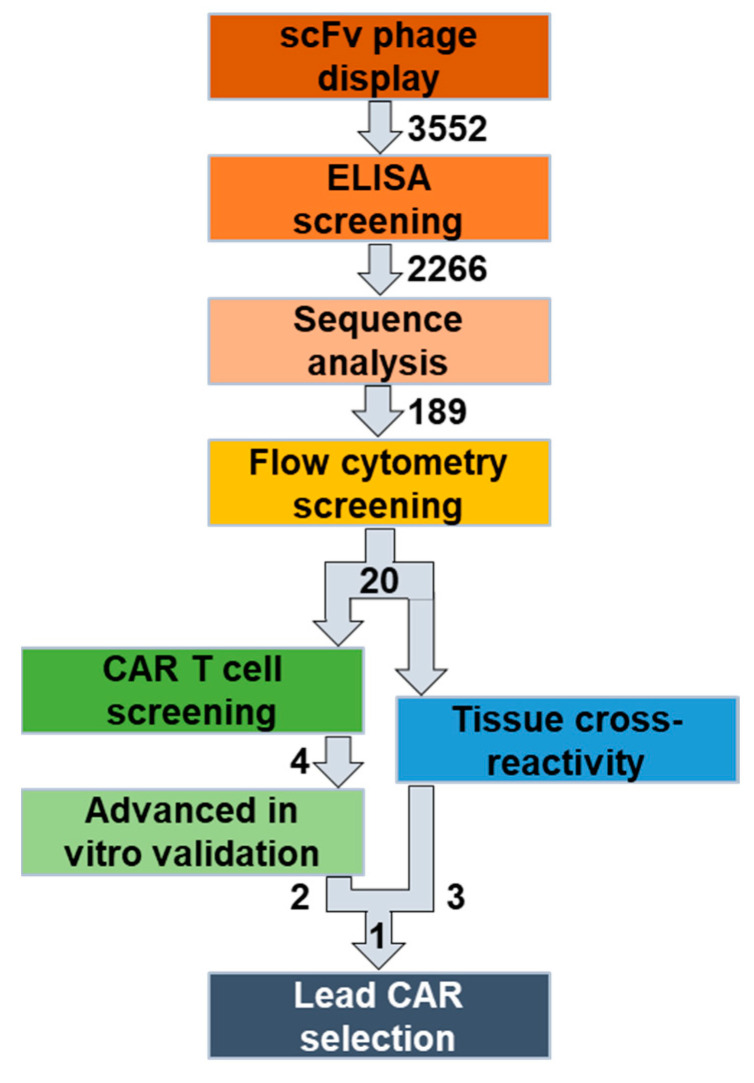
Overview of the identification and selection workflow for novel, fully human CAR T cell lead candidates. Initial panning and ELISA screening of two naïve human scFv phage display libraries resulted in >2266 candidates. After sequence analysis, 189 human/mouse cross-reactive anti-FOLR1 candidate binders were thereby identified. Flow cytometric screening of the binder candidates against human/murine FOLR variant-expressing cells was performed to select FOLR1-specific candidates. The 20 functional binders were characterized in two parallel approaches. In the first approach, the 20 functional binders were converted into CAR sequences and used to assess functionality, specificity, and cell expansion via a high-throughput CAR T cell screening approach. Finally, four candidates were selected for further advanced in vitro analysis. In the second approach, the 20 functional binders were used in a scFv-Fc format in a tissue cross-reactivity study employing ultra-high content imaging to assess potential on-target and off-target binding on human tissue samples. Finally, one anti-FOLR1 CAR lead candidate was selected based on the performance in both approaches.

To overcome these limitations, we established a novel workflow to identify and characterize novel, fully human CAR candidates (Figure 1). Here, we selected folate receptor 1 (FOLR1 or FRα) as the target antigen for the treatment of ovarian cancer [35,36]. We screened a naïve, human B cell receptor library to generate fully human scFv sequences specific for human and murine FOLR1, to be able to anticipate potential off-tumor reactivity in mouse studies. Since the folate receptor family has a high degree of similarity [37], we assessed specificity of the new human/murine FOLR1-directed binders towards other human/murine folate receptor variants hFOLR2, hFOLR3, hFOLR4, mFOLR2, and mFOLR4, respectively.

During the initial screening and panning campaigns, we performed positive and negative selections to isolate FOLR1-specific scFvs. Subsequently, FOLR1-binding domains were assessed as scFv-Fc fusion proteins in flow cytometry for advanced specificity characterization against members of the human and murine FOLR families. Next, FOLR1-specific binder sequences were transferred into second generation CAR constructs, and a functional in vitro high-throughput screening was performed, employing the FOLR1-proficient ovarian cancer cell line OV-90 as well as a CRISPR/Cas9-mediated FOLR1-deficient ovarian cancer cell line OV-90 *FOLR1* KO [36]. The high-throughput screening assay is based on a co-culture of CAR T cells with target cells and repetitive addition of fresh target cells. Automated plating of target cells and CAR T cells enabled the parallel analysis of all the CAR constructs simultaneously. The best functional performance of CAR candidates in vitro was determined by target cell lysis, cytokine release, and CAR T cell expansion employing automation approaches. Subsequently, FOLR1-targeting CAR candidates were further characterized in in vitro co-culture experiments under more challenging conditions, i.e., unfavorable effector to target cell ratios as well as repetitive target cell exposure. Finally, different ovarian cancer cell lines were employed as target cells in in vitro co-culture experiments, namely OV-90, OV-90 *FOLR1* KO, OVCAR-3, and SKOV-3.

In order to complement the anti-FOLR1 scFv characterization, we set up tissue cross-reactivity experiments employing a multiplexing imaging system. We used anti-FOLR1 scFv-Fc fusion proteins in an automated, high-plex imaging approach to analyze the respective staining profiles relative to a well-characterized anti-FOLR1 monoclonal antibody, LK26 [38,39], and to assess on-tumor and off-tumor binding on healthy human tissues (adrenal gland, ovary, pancreas, thyroid, cerebellum, cerebrum, lung, spleen, uterus, cervix, breast, placenta, heart, skin, skeletal muscle, kidney, stomach, small intestine, liver, and salivary gland) and malignant human tissues (high grade serous ovarian cancer).

Taken together, we established a new workflow to identify and characterize novel, fully human and functional CAR candidates against FOLR1, based on functional as well as tissue reactivity data.

## 2. Materials and Methods

Catalog numbers and company names of reagents are listed in Supplementary Table S3.

### 2.1. Identification of Antibody Fragments

Two human, naïve scFv phage display libraries, constructed at Miltenyi Biotec, were used for the identification of novel binders against FOLR1. These libraries differed in the variable domains of the light chain, as they contained either the κ or the λ isotype. The library was constructed from naïve human B cells of more than 120 healthy donors, resulting in an antibody diversity of 5 × 10^10^. For identification of antibody fragments, mouse FOLR1 (mFOLR1), human FOLR1-hIgG1Fc (hFOLR1-Fc), and human FOLR2 (hFOLR2) were purchased from Acro Biosystems. Human FOLR3 was purchased from R&D systems, and human IgG1-Fc (hIgG1Fc) was purchased from amsbio. For panning, antigens were directly immobilized on 96-well MaxiSorb ELISA Plates (Nunc) for 1 h at RT, followed by blocking with 1% (*w*/*v*) BSA in PBST (PBS supplemented with 0.05% Tween 20) for 1 h at RT. For each strategy, the κ and λ library was preincubated in BSA blocked wells to remove unspecific binding antibody phage particles, before incubation for 2 h on immobilized antigens of interest. Competing antigens were added directly after transferring the library to the antigen of interest. After stringent ELISA washing, scFv phage particles were eluted by adding 10 µg/mL trypsin and incubating for 30 min at 37 °C. Eluted phage particles were used to infect *E. coli* TG1 (O.D.600 0.4–0.5) and phage production was facilitated using M13K07 helper phage. After the third panning round, eluted phage particles were used to infect *E. coli* TG1 (O.D.600 0.4–0.5). Resulting single cell colonies were used to inoculate 96 well PP microtiter plates (Greiner BioOne, Kremsmuenster, Austria). The next day, 10 µL cultures were used to inoculate plates for expression of soluble scFv antibody fragments.

Expressed scFvs were analyzed for their capability to bind to hFOLR1-Fc, mFOLR1, hFOLR2, or hIgG1Fc in ELISA. A total of 30 ng antigen per well was coated using 384 well high binding PS microtiter plates (Greiner BioOne, Kremsmuenster, Austria), in PBS over night at 4 °C, and subsequent blocking was performed with 2% BSA-PBST (2% (*w*/*v*) BSA in PBST) for 1 h at RT. After three washing steps using an ELISA washer (EL406, BioTek), antibodies containing overnight cultures mixed with anti c-myc-HRP detection antibodies (Miltenyi Biotec, Bergisch Gladbach, Germany) were incubated for 1 h at RT. Finally, 30 µL of TMB (3,3′,5,5′-tetramethylbenzidine) was added and the color reaction was stopped by adding 30 µL 1 N sulfuric acid. Absorbances were measured at 450 nm (with 630 nm reference wavelength) using an ELISA reader (EPOCH, BioTek, Winooski, VT, USA).

### 2.2. Cloning of scFv-Fc Fusion Proteins

ScFv genes were cloned as scFv-Fc fusion into mammalian expression vector, based on pFUSE, InvivoGen (San Diego, CA, USA). Therefore, the scFv gene was amplified via PCR out of the phage display vector. ScFv genes and backbone vector were digested with NcoI/NotI and the backbone vector was additionally digested with Shrimp Alkaline Phosphatase (New England BioLabs, Ipswich, MA, USA) to avoid religation. NEB^®^ Stable Competent *E. coli* (High Efficiency) was used for transformation of the ligation mix. ScFv sequences were validated by Sanger sequencing.

### 2.3. Expression and Purification of Anti-FOLR1 scFv-Fc Candidates

Selected binders were transiently expressed as antibody fragments in the scFv-Fc format and subsequently purified via Protein A. Purity was confirmed via SDS page and protein concentrations were determined using a microplate reader by measuring UV absorbance at 280 nm. Purified proteins were stored at −20 °C until further processing.

### 2.4. Tissue Processing for Microscopy

We recently detailed our tissue processing method [40]. Briefly, frozen embedded tissue samples were sectioned at 8 µm thickness using a Leica CM3050 cryostat, and mounted on Menzel SuperFrost Plus slides (Thermo Fisher Scientific, Waltham, MA, USA). For acetone fixation, sections were fixed in acetone at −20 °C for 3 min, then stored at −80 °C. On the day of use, slides were immersed in acetone at −20 °C for 10 min to thaw, then briefly air-dried. The appropriate MACSwell™ Imaging Frame was immediately mounted on each slide, and the specified initial volume of MACSima™ Running Buffer was added, as outlined in the MACSwell™ Imaging Frame data sheet. For PFA fixation, cryo-sectioned slices were stored directly at −80 °C. On the day of use, slides were incubated in a 4% PFA solution at room temperature for 10 min and washed three times with MACSima™ Running Buffer. After washing, the MACSwell™ Imaging Frame and initial MACSima™ Running Buffer volume were added per the data sheet instructions. A preliminary DAPI staining was then performed: the sample was stained for 10 min with a 1:10 dilution of DAPI staining solution (volume according to the working format of the MACSwell™ Imaging Frames). Following this, the DAPI solution was removed, and three washes with MACSima™ Running Buffer were conducted. Finally, the initial volume of MACSima™ Running Buffer was added to the sample.

### 2.5. Cyclic Immunofluorescence Staining with the MACSima Imaging Platform

We have previously reported the use of the MACSima™ Imaging Platform [40]. The MACSima™ Imaging System is a fully automated instrument that integrates liquid handling with widefield microscopy for cyclic immunofluorescence imaging. Briefly, each staining cycle involves automated steps of immunofluorescent staining, sample washing, multifield imaging, and signal removal (either through photobleaching or REAlease Reagents). Cyclic immunofluorescence with the MACSima™ Imaging System is ideally performed on thin cryosections (a few microns thick), cultured cells, or suspension cells (either captured in microcavities or centrifuged onto glass). To enable MACSima™ imaging cyclic staining (MICS) experiments, we developed MACSwell™ Sample Carriers, which provide reaction cavities for the MACSima™ Imaging Platform. We also designed various sample carriers—MACSwell™ One, Two, and Four Imaging Frames, as well as MACSwell™ 24 Imaging Plates—to accommodate tissue sections of different sizes or adherent cells.

### 2.6. PE Labeling of scFv-Fc

ScFv-Fc proteins were coupled to Succinimidyl 4-[N-maleimidomethyl]cyclohexane-1-carboxylate (SMCC)-activated PE (Agilent, Cat-No: PB31). Labeling was performed on the semi-automated cell separator MultiMACS™ (Miltenyi Biotec, Bergisch Gladbach, Germany) using MACS^®^ Protein A and G MicroBeads and MACS^®^ Columns (Miltenyi Biotec, Bergisch Gladbach, Germany), according to the manufacturer.

### 2.7. MACSima™ Data Analysis

Tumor marker FOLR1 expression was quantified on a single-cell level on primary ovarian cancer tissue. Image data sets were segmented using MACS^®^ iQ View Software version 1.3.0 (Miltenyi Biotec, Bergisch Gladbach, Germany) based on nuclei, i.e., DAPI signal, and epithelial cell membrane marker EpCAM, identifying individual cells. Single cells were analyzed for FOLR1 expression. To identify single cells, the DAPI channel was used to segment cell nuclei and a distinct area around the nuclei as the cytoplasm. All features (cell size, nucleus size, biomarker expression, etc.) were measured in all channels. The mean intensities of all channels were calculated from the extracted data. Using MACS^®^ iQ Views plot tab feature, scatter plots of the positive reference versus the candidate binders were plotted, and the mean intensity of the negative binder was used as a cut-off value to set gates on the mentioned plots. The aim was to detect specific signals on single cells and, in particular, cells that were co-stained with the positive reference and the candidate binder, i.e., the double positive cells.

### 2.8. Generation of CAR Plasmids

Plasmids encoding the CAR constructs were prepared using standard molecular biology and cloning techniques. Human naïve single-chain variable fragments (scFv), identified after panning, were cloned into a lentiviral vector (Miltenyi Biotec, Bergisch Gladbach, Germany) using AscI and EcoRV. Construct configuration was scFv (Vh-Vl orientation)—human CD8alpha hinge—CD8alpha transmembrane domain—human 4-1BB costimulatory domain—human CD3 zeta domain. The scFv contained a (G4S)3 linker domain. Lentiviral vectors contained a truncated human low-affinity nerve growth factor receptor (ΔLNGFR) expression cassette separated by a P2A element for co-expression as a transduction marker.

### 2.9. Generation of CAR T Cells

The generation of CAR T cells has been previously described [41]. In summary, peripheral blood mononuclear cells (PBMCs) were isolated from whole blood samples via density gradient centrifugation. T cells were purified from PBMCs using the Pan T Cell Isolation Kit, human (Miltenyi Biotec, Bergisch Gladbach, Germany), and activated in TexMACS™ Medium (Miltenyi Biotec, Bergisch Gladbach, Germany) supplemented with T Cell TransAct™ (Miltenyi Biotec), along with 12.5 ng/mL of recombinant human interleukin IL-7 and 12.5 ng/mL of recombinant human IL-15 (both from Miltenyi Biotec, Bergisch Gladbach, Germany). Twenty-four hours after activation, T cells were transduced at a multiplicity of infection (MOI) of 5 using vesicular stomatitis virus glycoprotein G (VSV-G) pseudotyped lentiviral supernatants. Lentiviral particles were produced in-house by providing the required viral genes (gag/pol, rev and VSV-G) in trans, which was performed by co-transfection of 293T producer cells with four plasmids. Three days post-activation, T Cell TransAct™ was removed from the medium, and the cells were cultured in TexMACS™ Medium containing 12.5 ng/mL each of recombinant human IL-7 and IL-15. After an additional 9 to 12 days, CAR T cells were used for in vitro assays. On the day of assay use, the number of viable CAR T cells was determined by staining them with 7-AAD, biotinylated human FOLR1 Protein (Miltenyi Biotec, Bergisch Gladbach, Germany), and an anti-biotin antibody conjugated to PE.

### 2.10. Cell Lines and Culture Conditions

Human embryonic kidney 293T cells (HEK293T, ACC 635) were obtained from the DSMZ—German Collection of Microorganisms and Cell Cultures. OV-90 (CRL-11732), NIH:OVCAR-3 (HTB-161), and SKOV-3 (HTB-77) cells were obtained from ATCC and cultured as recommended. Jurkat E6 cells were cultured at 37 °C, 5% CO_2_ in RPMI with 10% FCS and 5 mM L-glutamine. Cell lines were routinely analyzed for mycoplasma contamination.

### 2.11. Cell Line Generation

OV-90 *FOLR1* KO cells were created using CRISPR/Cas9 followed by single-cell cloning. Ribonucleoprotein complexes were prepared according to the manufacturer’s instructions (IDT, Coralville, IA, USA). Briefly, CRISPR-Cas9 gRNA targeting FOLR1 (sequence: CCTACCTATATAGATTCAACTGG) was combined with CRISPR-Cas9 crRNA in a 1:1 ratio, incubated for 5 min at 95 °C, then, allowed to cool at RT for 20 min to form the gRNA complex. Next, the gRNA complex was incubated with Cas9 nuclease at a 1:1 ratio for 20 min at RT to form ribonucleoprotein complexes. Subsequently, 1 × 10^6^ OV-90 cells were resuspended in 40 µL of electroporation buffer (Miltenyi Biotec, Bergisch Gladbach, Germany), including 10 µL of ribonucleoprotein complexes (2.7 µg total), and transferred to an electroporation cuvette (Miltenyi Biotec, Bergisch Gladbach, Germany). Electroporation was performed using the CliniMACS^®^ Electroporator (Miltenyi Biotec). Afterward, cells were cultured under standard conditions, and *FOLR1* KO cells were sorted with the MACSQuant^®^ Tyto^®^ Cell Sorter (Miltenyi Biotec, Bergisch Gladbach, Germany). Knock-out efficiency was assessed through flow cytometry, qPCR, microscopy, and gDNA sequencing to confirm FOLR1 deletion. Single-cell clones were obtained by serial dilution.

Jurkat cells expressing various FOLR variants were generated via lentiviral transduction. Lentiviral particles encoded human FOLR1 (gene ID: 2348), human FOLR2 (gene ID: 2350), human FOLR3 (gene ID: 2352), human FOLR4 (gene ID: 390243), murine FOLR1 (gene ID: 14275), murine FOLR2 (gene ID: 14276), or murine FOLR4 (gene ID: 64931), respectively.

### 2.12. Cytotoxicity Assays

Target cells were seeded in triplicate in 96-well plates at densities of 2 × 10^4^ ovarian cancer cells, or 1.5 × 10^4^ Jurkat cells per well, in their respective culture media. CAR T cells were added 18 h later at an effector-to-target (E:T) cell ratio of 2:1, with the final well volume adjusted to 200 μL. The total T cell count was matched to that of the CAR T cell group with the lowest transduction efficiency. Cytotoxicity was assessed by monitoring the reduction in green surface area using the IncuCyte^®^ S3 Live-Cell Analysis System (Sartorius, Gottingen, Germany) and the associated IncuCyte^®^ software (versions v2017A, v2018C, and v2019A). The decrease in green surface area was calculated relative to baseline. At the end of the 48 h co-culture period, CAR T cells were analyzed by flow cytometry for activation markers (CD25, CD69, 4-1BB), exhaustion markers (TIM3, LAG3, PD1), and phenotype markers (CD62L, CD45RO, CD45RA). Additionally, after 24 h, 50 μL of medium was collected for cytokine analysis using the MACSPlex Cytokine 12 Kit, human (Miltenyi Biotec, Bergisch Gladbach, Germany). Then, 2 × 10^4^ GFP-expressing cells were seeded and co-cultured with 1 × 10^4^ anti-FOLR1 CAR T cells to measure antigen-dependent lysis of ovarian cancer cells for five days. After 48 h of co-culture, 2 × 10^4^ ovarian cancer cells were added to the co-culture. Cell lysis by the CAR T cell candidates was tracked by measurement of the green area confluency. As negative controls, ovarian cancer cells were cultured without addition of CAR T cells or with untransduced T cells. A positive control CAR T cell construct, known to successfully induce FOLR1-specific lysis of target cells, was included. The top four candidates identified by the CAR T cell screening were included in this assay, comprising three donors each. After 48 h and 108 h, flow cytometric measurement of activation marker expression 4-1BB and CD69 was performed. In addition, activation markers on a fourth donor were measured upon co-culture with FOLR1^low^ (OVCAR-3)-expressing cells.

### 2.13. Repetitive Killing Assays

Lentiviral particles were used to transduce primary human T cells of three independent donors for CAR expression with an MOI of 5. Frequency and absolute numbers of CAR T cells were determined by quantification of CD3^+^ LNGFR^+^ events using flow cytometry. CAR T cells were cultured at an E:T ratio of 2.5:1 with 2 × 10^4^ OV-90 or OV-90 *FOLR1* KO cells, which were transduced to express GFP. As controls, CAR T cells were cultured in absence of target cells, and OV-90 or OV-90 *FOLR1* KO cells were cultured in absence of CAR T cells. Additionally, OV-90 and OV-90 *FOLR1* KO cells were cultured with untransduced T cells or with T cells expressing one of two positive control CAR constructs, respectively. After 24 h of cell culture, supernatants were analyzed for cytokine secretion, e.g., IFN-γ, by using the MACSPlex Cytokine 12 Kit, human (Miltenyi Biotec, Bergisch Gladbach, Germany). After 92 h, CAR T cells (CD3^+^ LNGFR^+^) were quantified by flow cytometry. In addition, 2 × 10^4^ fresh OV-90 and OV-90 *FOLR1* KO cells were added, according to the experimental setup, to initiate the second round of co-culture. Twenty-four hours later, supernatants were again analyzed for IFN-γ secretion. After 164 h, 5 × 10^4^ CAR T cells (CD3^+^ LNGFR^+^) were quantified by flow cytometry. The third round of co-culture was started by addition of fresh OV-90 and OV-90 *FOLR1* KO cells, according to the experimental setup. Twenty-four hours later, supernatants were again analyzed for IFN-γ secretion. After 260 h, CAR T cells (CD3^+^ LNGFR^+^) were quantified by flow cytometry. The total duration of three rounds of co-culture was 11 days, and cytotoxicity towards OV-90 and OV-90 *FOLR1* KO cells, respectively, was quantified over time every 2 h by acquisition of the GFP signal over time by measuring the Total Integrated Intensity as Green Calibrated Units per µm^2^/image (IncuCyte S3, Sartorius, Gottingen, Germany).

### 2.14. Flow Cytometry

Target specificity of selected anti-FOLR1 scFv-Fc candidates was validated in flow cytometry using cell lines that were transduced with the respective FOLR variant. For multiplexing, non-target cells were labeled with CellTrace™ Violet or Cell Trace™ FarRed (ThermoFisher, Waltham, MA, USA) according to manufacturer’s protocol. Next, 1 × 10^5^ cells per cell line were seeded into a 96-well plate and cells were stained with 1 µg scFv-Fc for 30 min at 4 °C. Upon two washing steps, secondary antibody staining was performed using AffinitPure F(ab’)_2_ Fragment Goat Anti-Human IgG-PE (Jackson ImmunoResearch, Cambridgeshire, UK) for 10 min at 4 °C.

Antibody conjugates that were used for surface marker expression are listed in Supplementary Table S3. Stainings performed with antibody conjugates from Miltenyi Biotec (Bergisch Gladbach, Germany) were performed as recommended by the supplier. Antibodies from Biolegend were applied at a concentration of 5 µg/mL and incubated for 10 min at 4 °C, followed by a successive washing step. To exclude dead cells, 7-AAD or PI was used. Samples were acquired at the MACSQuant^®^ Analyzer 10 or 16. MACSQuantify™ Software v2.13.0/v2.11.0, and Flow Logic software 7.2.1 were used for data analysis.

For quantification of CAR T cell expansion in repetitive killing assays, samples were taken at the end of each round of co-culture. After incubation with antibodies in PEB (CliniMACS^®^ PBS/EDTA buffer supplemented with 0.5% MACS^®^ BSA stock solution), the cell suspension was diluted by the addition of PEB plus Poloxamer 188 solution. These samples were acquired on a MACSQuant^®^ X Flow Cytometer (Miltenyi Biotec, Bergisch Gladbach, Germany).

### 2.15. Statistical Analysis

Unless indicated otherwise, all graphs display the mean, with error bars representing the standard error of the mean. Statistical analyses were performed with GraphPad Prism 8.

### 2.16. Primary Human Blood and Tissues

High-grade serous ovarian cancer patient samples were purchased from BioIVT or provided by Prof. Dr. Peter Mallmann (Department of Obstetrics and Gynaecology, Faculty of Medicine, University of Cologne), with all samples obtained under informed patient consent. FDA Standard Frozen Tissue Array—Human Adult Normal was purchased from Biocat (Heidelberg, Germany), including adrenal gland, ovary, pancreas, thyroid, cerebellum, cerebrum, lung, spleen, uterus, cervix, breast, placenta, heart, skin, skeletal muscle, kidney, stomach, small intestine, liver, and salivary gland.

### 2.17. Ethics Approval

For all studies involving human primary ovarian cancer tissue, written informed consent was obtained in accordance with the University Hospital Cologne Review Board’s approved protocol. Healthy whole blood samples were collected from donors who had provided prior written consent. Peripheral blood mononuclear cells (PBMCs) were isolated from buffy coats obtained from anonymous healthy donors, purchased from the German Red Cross in Dortmund. All blood samples were handled following required ethical and safety guidelines.

## 3. Results

### 3.1. Alternating Selection Strategy Identifies Unique, Fully Human Binder Sequences Against Human and Murine FOLR1

**Figure 2:**
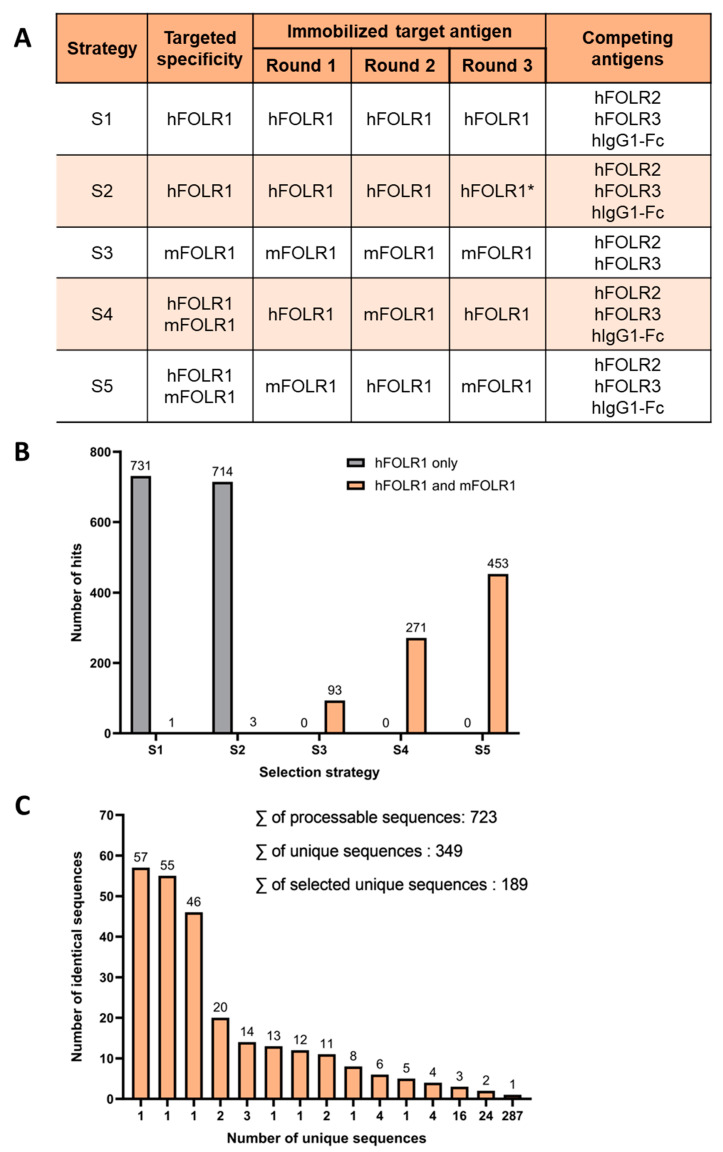
Phage display reveals unique binder sequences with cross-reactivity for hFOLR1 and mFOLR1. (**A**) Five different panning strategies were used to identify binder candidates using hFOLR1 and mFOLR1 antigens for positive selection, respectively. In all strategies, hFOLR2, hFOLR3, and hIgG1-Fc (except in Strategy S3) were used for competition. Strategy 2 used a lower amount of antigen in the third panning round (indicated by *) compared to strategy 1. (**B**) Quantification of positive clones in each panning strategy confirms that the five different selection strategies lead to the discovery of scFvs that bind preferably to the target(s) that were used in the panning strategies, e.g., panning strategies 4 and 5 resulted in scFvs binding preferably to both hFOLR1 and mFOLR1 antigen. (**C**) Sequencing of scFv hits confirmed 349 unique sequences derived from 723 processable sequences of panning strategies 3–5.

Two naïve human scFv phage display libraries containing either kappa- or lambda-light chains were employed for the identification of novel binders, cross-reactive against human and murine FOLR1 (hFOLR1 and mFOLR1, respectively). Five different panning strategies were applied to identify binder candidates using hFOLR1 and mFOLR1 antigen for positive selection, either alone or in combination, to facilitate the selection of cross-reactive binders (Figure 2A). Strategies using either hFOLR1 or mFOLR1 alone were employed to determine whether a single antigen is sufficient to generate cross-reactive binders. For all approaches, a combination of hFOLR2, hFOLR3, and hIgG1-Fc antigens was used for competition, except in strategy S3, where only hFOLR2 and hFOLR3 were used for competition.

### 3.2. Flow Cytometric Screening Identifies a Total of 20 Anti-FOLR1 scFv-Fc Candidates That Specifically Recognize Both hFOLR1 and mFOLR1 Expressed by Jurkat Cells

After panning, scFvs were analyzed for their capability to bind to hFOLR1-Fc, mFOLR1, hFOLR2, or hIgG1-Fc by ELISA. This led to the identification of 2266 hits that bound either to hFOLR1 and/or mFOLR1 and did not bind to hFOLR2 or hIgG1-Fc. As expected, cross-reactive scFvs were preferably identified from approaches, where both hFOLR1 and mFOLR1 were used in the selection, i.e., S4 and S5 (Figure 2B and Figure S1). On the other hand, panning strategy S3, with selection against mFOLR1, revealed scFv candidates binding preferably to mFOLR1 over hFOLR1 as expected, but also led to a decent number of cross-reactive clones (Figure 2B and Figure S1). Panning strategies using both hFOLR1 and mFOLR1 as target antigens, i.e., strategies S4 and S5, led to the selection of scFvs that were cross-reactive against both targets (Figure 2B and Figure S1). Out of the 2266 first hits, 817 cross-reactive scFv clones were sequenced. From these 817 sequences, the sequences containing stop codons, truncated sequences, or sequences with low sequencing quality were excluded, resulting in 723 sequences. Alignment of the 723 scFv sequences revealed 349 unique sequences (Figure 2C). From the unique sequences, 189 sequences were chosen, based on their abundance, for further processing (Supplementary Figure S1).

Taken together, we applied multistep panning and phage display strategies including alternating target antigen selection, i.e., human and murine FOLR1, as well as negative selection with several competing proteins. Thereby, we selected 189 unique human binder sequences recognizing human and/or murine FOLR1 for further cellular screening by flow cytometry.

**Figure 3:**
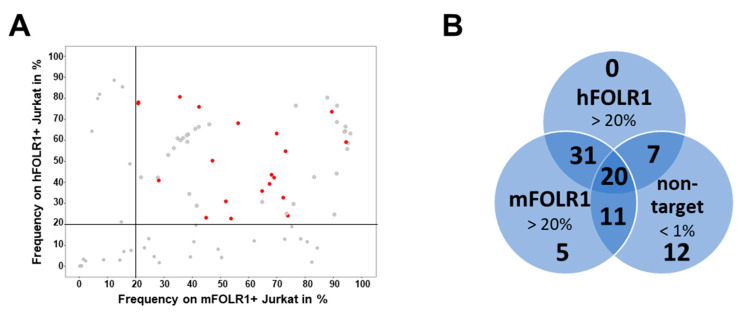
Flow cytometric analysis of binders in the scFv-Fc format identifies candidates enabling specific staining of FOLR1-expressing cells compared to other FOLR variants. (**A**) Following generation of candidates in scFv-Fc format, binders were validated on Jurkat cells expressing hFOLR1 or mFOLR1, respectively. Flow cytometric measurements were performed in triplicates and are displayed as mean frequency of stained cells. Lead candidates are marked in red. Binders that do not meet the selection criteria are marked in grey. (**B**) Venn diagram indicating the number of clones meeting the respective criteria for each cell line staining. Staining of hFOLR1- and mFOLR1-expressing Jurkat cells was considered positive if frequencies >20% were detected. Staining of non-target cells expressing either hFOLR2, mFOLR2, hFOLR4, or mFOLR4, respectively, was considered negative if frequencies <1% were detected. In total, 87 binders were analyzed.

To characterize the novel anti-FOLR1 binder candidates in a cellular context, scFvs were expressed as scFv-Fc fusion proteins and validated by flow cytometric analysis using cell lines that express different human and murine FOLR proteins (Supplementary Figure S2A). From the 189 hits, 86 were expressible as scFv-Fc proteins in HEK cells. First, we analyzed 86 scFv-Fc candidates regarding their staining performance on transgenic target cells, including both hFOLR1- and mFOLR1-expressing Jurkat cells. Staining frequencies of >20% on hFOLR1- and mFOLR1-expressing Jurkat cells were detected for 58 and 67 anti-FOLR1 scFv-Fc candidates, respectively (Figure 3A). Of those, 51 anti-FOLR1 scFv-Fc candidates showed positive staining for both hFOLR1- and mFOLR1-expressing Jurkat cells and are, thus, considered as cross-reactive (Figure 3A,B). Second, we analyzed off-target binding of all 86 scFv-Fc candidates to non-target cells, i.e., FOLR2- and FOLR4-expressing Jurkat cells and wildtype Jurkat cells. Out of 86 scFv-Fc candidates, 36 candidates showed unspecific staining, defined as staining frequency > 1% on hFOLR2- and mFOLR2-expressing Jurkat cells, whereas no off-target staining was detected for hFOLR4- and mFOLR4-expressing Jurkat cells, as well as wildtype Jurkat cells (Supplementary Figure S2B,C).

In total, 20 candidates were selected for further analysis in CAR T cell assays based on specific binding to both human and mouse FOLR1-expressing Jurkat cells. Candidates binding to h/mFOLR2- or h/mFOLR4-expressing Jurkat cells were excluded from further analysis (Figure 3B).

### 3.3. High-Throughput Screening Enables Functional Anti-FOLR1 CAR T Cell Screening In Vitro

**Figure 4:**
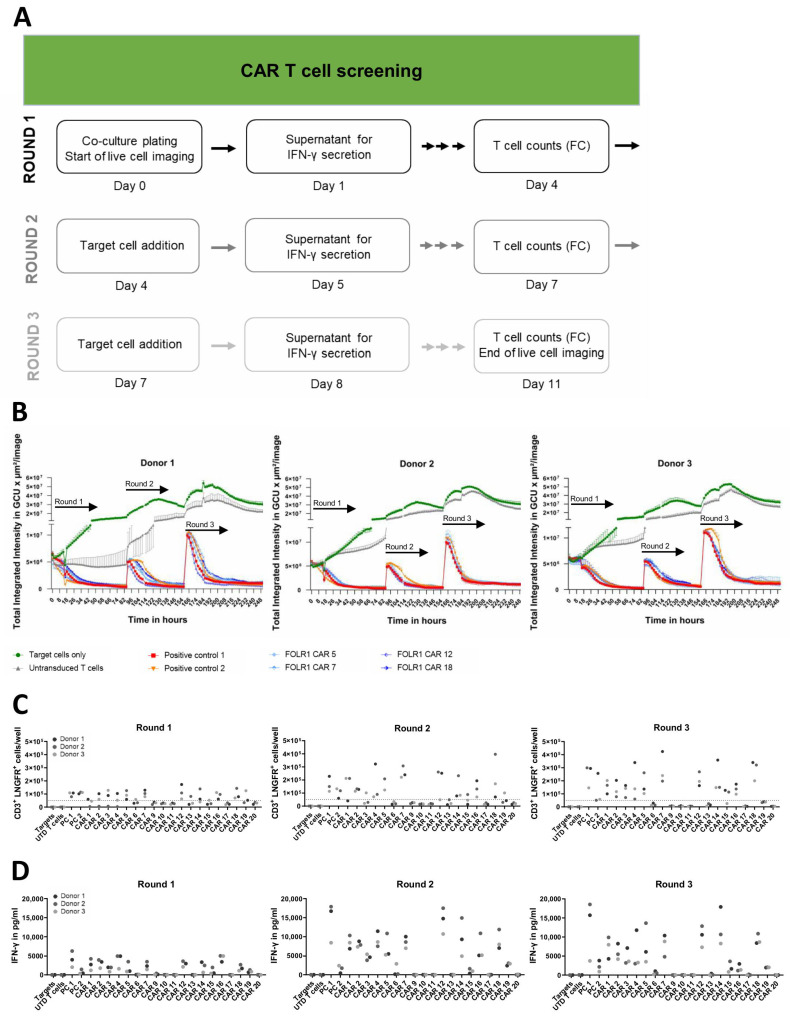
CAR T cell screening reveals functional, FOLR1-specific CAR T cell candidates in vitro. (**A**) Workflow scheme to identify functional and specific CAR T cells in serial in vitro co-culture assays. Read-outs included real-time live cell analysis, quantification of cytokine secretion after repeated addition of target cells, and flow cytometric quantification of transduced T cells at the end of each round of co-culture. (**B**) Lysis of ovarian cancer cells (OV-90) expressing FOLR1 by four CAR candidates of three different donors. GFP-expressing OV-90 target cells were seeded and co-cultured with anti-FOLR1 CAR T cells to measure antigen-dependent lysis of ovarian cancer cells. After 92 h of co-culture, fresh OV-90 cells were added, followed by a second addition of OV-90 cells after 164 h. Target cell lysis by the CAR T cell candidates was analyzed by decrease in GFP signal over time by measuring the Green Calibrated Units (GCU) per µm^2^/image for 11 days. As negative controls, OV-90 target cells were either cultured without addition of CAR T cells or co-cultured with untransduced T cells. Two positive control CAR T cell constructs, known to successfully induce FOLR1-specific lysis of target cells, were included. Error bars indicate standard deviation (SD) of respective mean values. (**C**) CAR T cell expansion of all candidates from three different donors as CD3^+^ LNGFR^+^ cells/well after 92 h, 164 h, as well as 260 h of co-culture, respectively. Cell counts were determined by flow cytometry before each addition of target cells, and at the endpoint, respectively. Dotted line indicates 50,000 transduced T cells used as CAR T cell input on day 0. No significant differences were found amongst CAR T cell preparations at the end of round 1 and 2. However, at the end of round 3, anti-FOLR1 CARs 6, 9, 10, 11, 13, 17, 19, and 20 resulted in significantly lower proliferative responses compared to positive control 1. (**D**) IFN-γ secretion in repeated co-cultures 24 h after each addition of OV-90 cells to candidate CAR T cells. No significant differences in IFN-γ secretion amongst CAR T cell preparations were observed for anti-FOLR1 CARs 1, 2, 4, 5, 7, 12, 14, 16, and 18 compared to positive control 1 in round 1. All other CAR T samples expressed significantly lower IFN-γ. In round 2, amounts of IFN-γ secreted by anti-FOLR1 CAR T cells 4, 12, 14, and 18 were not significant compared to positive control 1. All other CAR T cell preparations produced significantly lower IFN-γ. Finally, in round 3, no differences were identified for anti-FOLR1 CARs 1, 2, 4, 5, 7, 12, 14, and 18, whereas all other CAR constructs mediated significantly lower cytokine production by T cells. Statistical analysis was performed by Dunnett post hoc analysis after 1-way ANOVA. Abbreviations in figure: untransduced (UTD) and positive control (PC).

Nineteen of the nominated twenty scFv candidates selected from the flow cytometric analysis were successfully cloned into a second-generation CAR backbone for functional assessment. CAR T cells were generated by transduction at MOI 5, and transduction efficiencies ranged from 5 to 21% (mean 13.5%, donor 1), 6 to 26% (mean 14.8%, donor 2), and 3 to 26% (mean 9.7%, donor 3). CAR T cells were expanded without enrichment or sorting. Three consecutive rounds of co-culture of CAR T cells with OV-90 target cells allowed for the identification of CAR candidates, which were able to mediate repeated killing of FOLR1-expressing cells as an in vitro surrogate approach for effective reduction of tumor burden (Figure 4A). Functionality of CAR candidates was confirmed in co-cultures of OV-90 cells with CAR T cells of two additional donors, as exemplified by anti-FOLR1 CAR candidates 5, 7, 12, and 18 (Figure 4B). These anti-FOLR1 CAR candidates were selected for a focused graphical representation as they also appeared to be functionally superior over other anti-FOLR1 CAR candidates in additional readouts. Thus, the experimental setup allowed for the discrimination of non-functional binders in CAR format from those that mediated cytotoxicity in one, two, or up to three rounds of co-culture (Supplementary Figure S3A). In addition, co-cultures of CAR T cells with OV-90 *FOLR1* KO cells allowed for the identification of CAR candidates, which mediated cytotoxicity in the first and up to the third round of co-culture, in the absence of target antigen expression (Supplementary Figure S3B), i.e., antigen-independent cytolysis of anti-FOLR1 CAR candidate 16.

For the exemplary anti-FOLR1 CAR candidates 5, 7, 12, and 18, CAR T cell expansion was observed for all three donors in co-cultures with OV-90 cells at the end of the first and the third round, i.e., CAR T cell numbers were exceeding the initial amount of CD3^+^ LNGFR^+^ T cells plated at day 0 (Figure 4C). Expression of functional CAR constructs 5, 7, 12, and 18 by T cells was associated with secretion of IFN-γ, widely used as a potency measure for CAR T cells [42], in each round of co-culture with OV-90 cells (Figure 4D). Anti-FOLR1 CAR candidate 16, which induced T cell-mediated cytotoxicity in OV-90 *FOLR1* KO co-cultures, also induced IFN-γ secretion in each round of co-culture with OV-90 and OV-90 *FOLR1* KO cells, respectively, indicating off-target reactivity (Figure 4D and Figure S3C). In sum, in vitro co-culture experiments allowed for the selection of CAR T cell candidates with the ability to mediate repeated killing, cytokine production, as well as expansion upon target cell encounter while sparing target antigen deficient cells. Consequently, we selected anti-FOLR1 CAR candidates 5, 7, 12, and 18 for further in vitro assessment.

### 3.4. Advanced In Vitro CAR T Cell Assays Identify Functional Lead Anti-FOLR1 CAR T Cell Candidate

**Figure 5:**
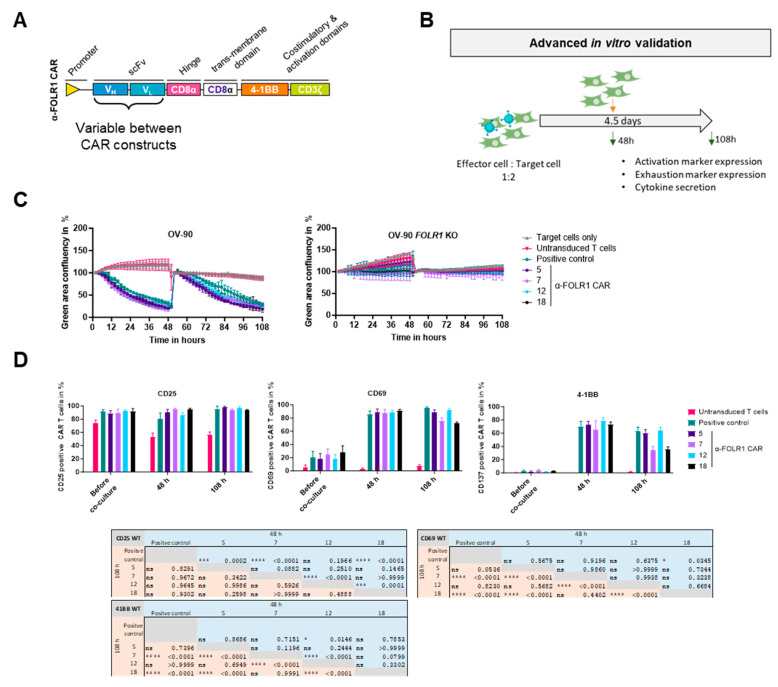

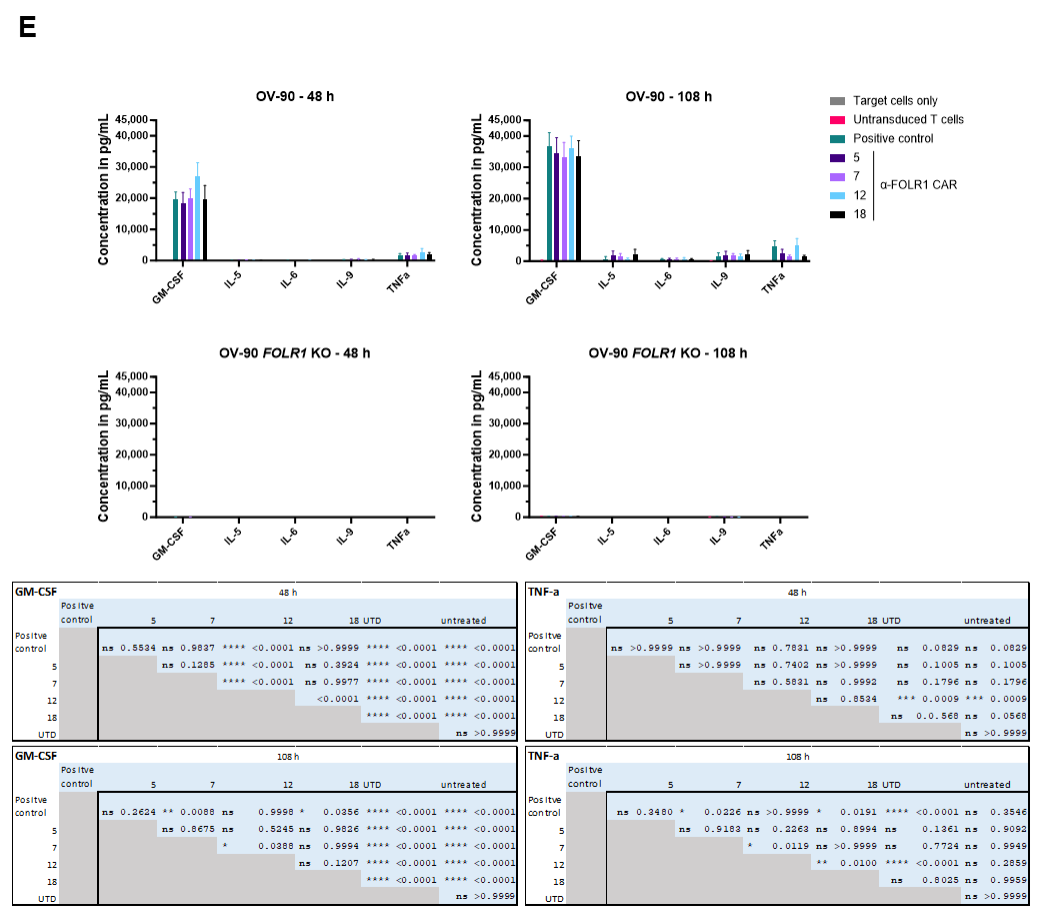
Advanced in vitro CAR T cell assays identify functional lead anti-FOLR1 CAR T cell candidate. (**A**) Schematic CAR architecture of the anti-FOLR1 CAR T cell candidates consisting of a FOLR1-directed scFv, CD8 hinge, CD8 transmembrane domain, 4-1BB co-stimulatory domain, and CD3ζ activation domain, respectively. (**B**) Workflow scheme of advanced in vitro assays. GFP-expressing ovarian cancer cells were seeded and co-cultured with anti-FOLR1 CAR T cells (effector cell-to-target cell ratio of 1:2) to measure antigen-dependent lysis of ovarian cancer cells for five days. After 48 h of co-culture, fresh ovarian cancer cells were added to the co-culture. Cell lysis by the CAR T cell candidates was measured by changes in the green area confluency. After 48 h and 108 h, respectively, activation and exhaustion marker expression were analyzed by flow cytometry and cytokine secretion was assessed with the MACSPlex Cytokine 12 Kit. (**C**) Representative killing assay of four anti-FOLR1 CAR candidates co-cultured with FOLR1-proficient, GFP-expressing ovarian cancer cells (OV-90, left) and FOLR1-deficient, GFP-expressing ovarian cancer cells (OV-90 *FOLR1* KO, right). Data points represent mean values and standard deviation (SD) is indicated (*n* = 3). (**D**) Expression of activation markers CD25, CD69, and 4-1BB at the indicated time points was analyzed by flow cytometry (three donors). Bars represent mean values and SD is depicted (*n* = 3). Statistical Analysis: Two-way ANOVA a = 0.05, ns *p* > 0.05, * *p* ≤ 0.05, ** *p* ≤ 0.01, *** *p* ≤ 0.001, **** *p* ≤ 0.0001. (**E**) Concentration of secreted cytokines in supernatant from co-culture with OV-90 cells at the indicated time points was analyzed with the MACSPlex Cytokine 12 Kit. Bars represent mean values, and SD is depicted (*n* = 3). Crosses indicate values above the upper detection limit.

The four CAR constructs encoding anti-FOLR1 CAR 5, 7, 12, and 18, respectively, were modified, i.e., by removing the LNGFR reporter gene, to adapt the CAR constructs to clinical requirements and maintaining the previously described second-generation CAR design (Figure 5A).

To validate functionality and specificity of the anti-FOLR1 CAR candidates, CAR T cells of three donors were co-cultured with GFP-expressing target cells (OV-90) or non-target cells (OV-90 *FOLR1* KO) at a modified initial effector-to-target cell ratio of 1 to 2, in contrast to the previous effector-to-target cell ratio of 2 to 1. After 48 h, fresh target cells were added to the co-culture to re-challenge the respective anti-FOLR1 CAR T cells. CAR T cells were analyzed for activation as well as exhaustion marker expression by flow cytometry and co-culture supernatants were analyzed for secreted cytokines after 48 h and 108 h, respectively (Figure 5B). As controls, OV-90 cells were cultured either without T cells or with untransduced T cells. In addition, positive control anti-FOLR1 CAR T cells were co-cultured with OV-90 cells, known to specifically and efficiently induce lysis of FOLR1-expressing cells [36].

CAR candidates efficiently eradicated OV-90 cells expressing FOLR1, even after repeated addition of OV-90 cells, with a tendency to perform better than the positive control (Figure 5C (left panel) and Figure S4A (left panel)). Additionally, CAR candidates did not eradicate OV-90 *FOLR1* KO cells (Figure 5C (right panel) and Figure S4A (right panel)). These findings indicate very efficient CAR T cell candidates, as well as very specific CAR constructs under challenging conditions, i.e., low effector-to-target cell ratio and re-challenge with additional tumor cells.

After 48 h and 108 h, the expression of activation markers CD25, CD69, and 4-1BB was measured on the CAR T cells via flow cytometry. CAR T cells were prolonged and efficiently activated, as shown in increased CD25, CD69, and 4-1BB expression, compared to untransduced T cells (Figure 5D). Differences in the activation profiles between the four CAR candidates can be seen after 48 h of co-culture with OV-90 cells for CD25. While anti-FOLR1 CAR 12 expressed similar levels of CD25, anti-FOLR1 CAR 5, anti-FOLR1 CAR 7, and anti-FOLR1 CAR 18 showed increased CD25 expression levels compared to the positive control (Figure 5D, left). After 108 h, CD25 expression was comparable for all candidates cultured with FOLR1-proficient cells (Figure 5D, left).

Expression of activation marker CD69 was similar for all candidates cultured with OV-90 cells after 48 h (Figure 5D, middle). After 108 h, anti-FOLR1 CAR 7 and anti-FOLR1 CAR 18 showed reduced expression compared to anti-FOLR1 CAR 5 and anti-FOLR1 CAR 12, after co-culture with OV-90 target cells (Figure 5D, middle). However, CD69 expression of anti-FOLR1 CAR 7 and anti-FOLR1 CAR 18 was elevated compared to anti-FOLR1 CAR 5, anti-FOLR1 CAR 12, and the positive control CAR T cells, after co-culture with OV-90 *FOLR1* KO cells (Supplementary Figure S4B, middle).

Differences in the activation profiles between the four CAR candidates can be seen after 48 h of co-culture with FOLR1-proficient OV-90 cells for 4-1BB expression. Only anti-FOLR1 CAR 12 showed significantly increased expression of 4-1BB compared to the positive control. Only after 108 h, however, differences for 4-1BB became even more pronounced. Anti-FOLR1 CAR 7 and anti-FOLR1 CAR 18 showed reduced 4-1BB expression compared to the positive control, whereas anti-FOLR1 CAR 5 and anti-FOLR1 CAR 12 maintained high expression after 108 h (Figure 5D, right). 4-1BB activation marker expression of all candidates was not induced after co-culture and re-challenge with OV-90 *FOLR1* KO cells (Supplementary Figure S4B, right).

CAR T cell exhaustion was quantified by analyzing the expression of exhaustion markers (LAG3, PD1, TIM3) (Supplementary Figure S4C). The positive control and CAR candidate 12 revealed similar expression levels of all double exhaustion marker combinations, after 48 h of co-culture with FOLR1-proficient OV-90 cells. CAR candidates 5, 7, and 18 showed reduced exhaustion marker expression compared to the positive control, whereas untransduced T cells did not exhibit any exhaustion marker expression. After 108 h, however, expression levels for all marker combinations and CAR candidates, as well as for the positive control, were increased. CAR candidate 12 and the positive control had comparable marker expression, but higher expression levels compared to other anti-FOLR1 CAR candidates. Anti-FOLR1 candidates CAR 7 and CAR 18 showed the least exhaustion marker expression followed by anti-FOLR1 CAR 5. A low frequency of triple exhaustion marker-expressing cells was detected, only after co-culture with FOLR1-proficient OV-90 cells, after 48 h and 108 h, respectively. Only CAR candidate 12 showed an increased percentage of triple positive CAR T cells after 108 h. Upon co-culture with OV-90 *FOLR1* KO cells, neither the positive control nor the candidates exhibited exhaustion marker expression after 48 h or 108 h, respectively (Supplementary Figure S4C).

We performed an additional co-culture experiment using anti-FOLR1 CAR T cell candidates co-cultured with ovarian cancer cell lines with varying FOLR1 expression levels [36], i.e., OVCAR-3 (FOLR1^low^), SKOV-3 (FOLR1^intermediate^), and OV-90 (FOLR1^high^), as well as OV-90 *FOLR1* KO cells (Supplementary Figure S4D). Slightly increased CD25 expression was detected for anti-FOLR1 CAR 7, anti-FOLR1 CAR 12, and anti-FOLR1 CAR 18 compared to the positive control, after co-culture with OVCAR-3 FOLR1^low^ cells (Supplementary Figure S4D, left). CD69 and 4-1BB activation marker expression of CAR candidates was increased after 48 h of co-culture with OV-90 cells, while minimal activation of all CAR candidates was observed after co-culture with OV-90 *FOLR1* KO cells (Supplementary Figure S4D, middle and right). Anti-FOLR1 CAR 7 and anti-FOLR1 CAR 18 showed reduced activation marker expression (CD69 and 4-1BB) compared to anti-FOLR1 CAR 12 after co-culture with SKOV-3 and OVCAR-3 cells, respectively. Anti-FOLR1 CAR 5 expressed CD69 and 4-1BB only at a higher level compared to anti-FOLR1 CAR 7 and anti FOLR1 CAR 18 after co-culture with FOLR1^intermediate^- or FOLR1^high^-expressing cells. Interestingly, anti-FOLR1 CAR candidate 12 expressed higher levels of CD69 and 4-1BB after co-culture with FOLR1^low^-expressing OVCAR-3 cells. This may be indicative of a higher sensitivity of CAR candidate 12 towards cancer cells with low FOLR1 expression.

Moreover, we analyzed the co-culture supernatant for secreted cytokines (Figure 5E). After co-culture with OV-90 cells, anti-FOLR1 CAR candidate 12 secreted more GM-CSF and IL-2 after 48 h than the other candidates. After 108 h, anti-FOLR1 CAR candidate 12 secreted more GM-CSF and TNFα than the other candidates. Anti-FOLR1 CAR candidates 7 and 18 presented with the lowest concentrations of cytokines secreted. Noteworthy, after co-culture with OV-90 *FOLR1* KO cells, none of the anti-FOLR1 CAR candidates secreted detectable amounts of cytokines.

Taken together, all four tested CAR candidates were functional and specific in vitro. Anti-FOLR1 CAR candidates 5 and 12 revealed elevated expression of activation marker CD69 and 4-1BB after co-culture with FOLR1-expressing cells. Additionally, the expression of activation markers by anti-FOLR1 CAR candidates 5 and 12 tended to be reduced in cells lacking FOLR1, relative to the positive control anti-FOLR1 CAR. As anti-FOLR1 CAR 12 was strongly activated by FOLR1^low^-expressing target cells and showed increased cytokine secretion, consequently, anti-FOLR1 CAR 12 was nominated as lead candidate.

### 3.5. Tissue Cross-Reactivity Analysis Reveals Anti-FOLR1 Binder Candidates’ Off-Target Interaction Profile

In addition to the characterization of cellular functionality of CAR T cells, we also assessed reactivity of the FOLR1-targeting binders towards human tissues. For initial safety assessment we established a new tissue cross-reactivity workflow evaluating the binding specificity of the novel scFvs by identification of potential cross-reactivities of the scFv-Fc candidates. Employing scFv-Fc fusion proteins as staining reagents conserves the binding moiety of the CAR construct. This implies that no reformatting into an antibody is necessary, which may introduce changes into the binding moiety and, consequently, may alter the binding profile. Thereby, lead candidates can be selected early in the development process based on this safety-relevant feature.

**Figure 6:**
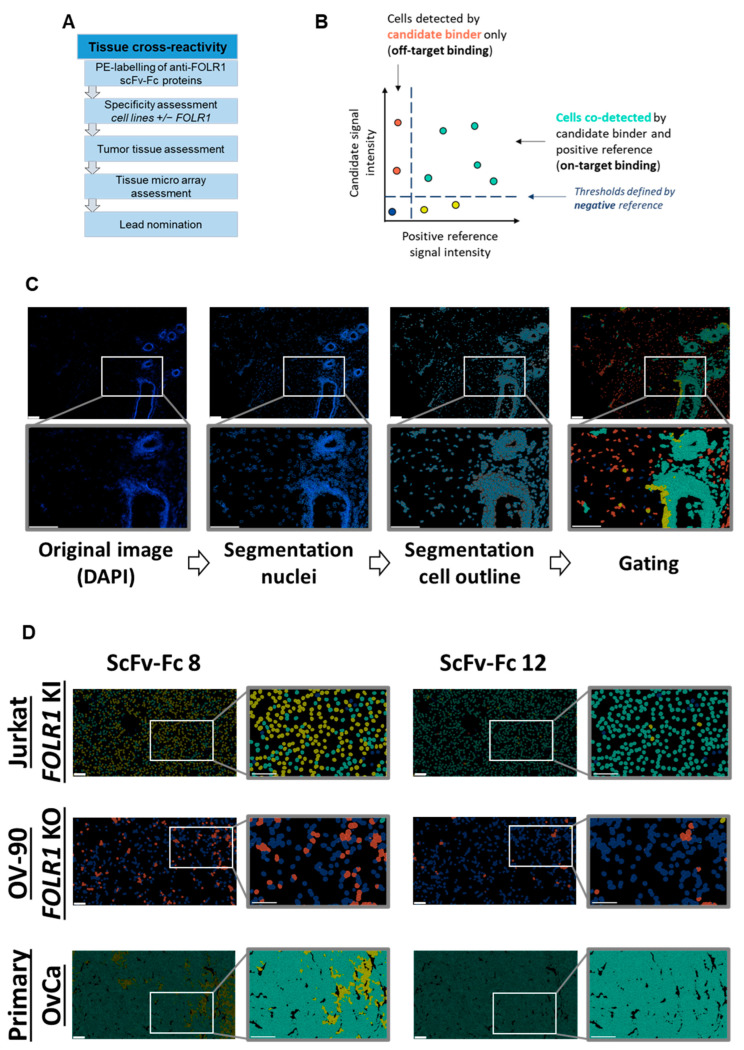

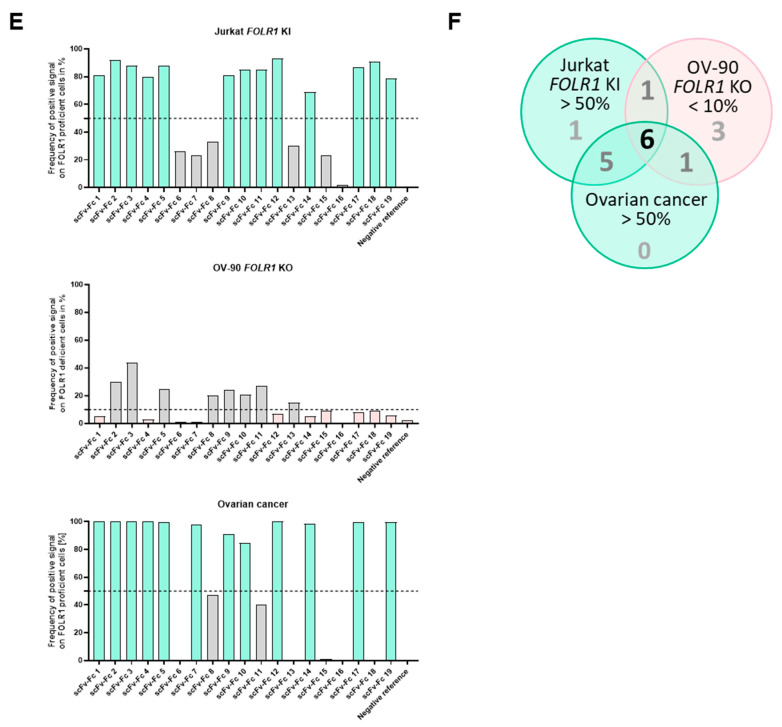
Tissue cross-reactivity workflow identifies anti-FOLR1 binder candidates’ on-/off-target and on-tumor binding profiles. (**A**) Overview of the tissue cross-reactivity workflow and the individual steps. (**B**) Gating strategy for on-/off-target binding analysis of new scFv-Fc binders. Thresholds are based on the mean intensity value of the negative reference. Cells color-coded cyan are defined as on-target binding, whereas red-labeled cells are defined as off-target binding. (**C**) Example of cell segmentation workflow for on-/off-target binding analysis according to gating strategy in (**B**). Nuclear borders identified from DAPI signal are marked in blue, whereas cell cytoplasms are filled in grey. The color-code for cell gating is adapted from gating strategy shown in (**B**). (**D**) Exemplary representation of cell segmentation and on-/off-target gating of binder candidates 8 and 12 (scFv-Fc 8 and scFv-Fc 12, respectively). The staining was performed on FOLR1-proficient cell lines (*FOLR1* knock-in (Jurkat *FOLR1* KI), top row), and FOLR1-deficient cell lines (FOLR1 knock-out (OV-90 *FOLR1* KO, middle row). Additionally, primary OvCa tissue (bottom row) was segmented, and binders were analyzed for on-tumor reactivity. (**E**) Quantification of the on- and off-target binding frequency of all binder candidates on cell lines (Jurkat *FOLR1* KI and OV-90 *FOLR1* KO, respectively) and primary OvCa tissue. Thresholds (dotted line) for evaluation of binder candidate specificity was set to >50% for on-target binding, and <10% for off-target binding. (**F**) Venn diagram summarizes on-target (>50% staining frequency of Jurkat *FOLR1* KI cells), off-target (<10% staining frequency of OV-90 *FOLR1* KO cells), and on-tumor reactivity (>50% staining frequency of tumor cells within primary ovarian cancer tissue) of new CAR binder candidates. Taken together, six candidates match the requirements. All scale bars represent 100 µm.

The tissue cross-reactivity workflow is a multistep process (Figure 6A). Nineteen out of twenty scFv-Fc candidates nominated from the flow cytometry screening were successfully conjugated to phycoerythrin (PE) and employed in cyclic immunofluorescence analysis on acetone- or PFA-fixed samples, using the automated MACSima™ platform. Cyclic immunofluorescence is based on iterative immunofluorescent staining, sample washing, multifield imaging, and signal erasure. Subsequent data processing allowed for the segmentation of the tissue images into single cells and to analyze cells according to their expression profiles [36,40,41,43]. Multiplexing on the same tissue sample allowed for analyzing and comparing the staining profiles, i.e., signal intensity and distribution, of novel antibody fragments in relation to reference antibodies targeting the same antigen. Here, the previously characterized anti-FOLR1 monoclonal antibody LK26 was used as a positive reference [44,45]. In addition, a negative staining reference was used to determine thresholds for positive and negative signals (Figure 6B). The mean intensity value of the negative reference was set at a lower threshold value, i.e., intensity values higher than the mean intensity value of the negative reference were considered as specific signals. Image segmentation allowed for the identification of individual cells in each sample, as well as determination of signal intensities of candidate binders and the positive control. Thereby, segmented cells could be gated and analyzed (Figure 6C). Subsequent correlation analysis of scFv-Fc candidates and the positive reference allowed for identification of on-target binding candidates. Staining of cells only by a scFv-Fc candidate was considered as off-target binding. Based on the described on- and off-target binding frequencies, we selected lead candidates.

To exclude unspecific candidates, binding of scFv-Fcs on cell lines with defined target expression, here, transgenic knock-in or CRISPR/Cas9-mediated knock-out of FOLR1, was analyzed. Next, binders were tested on primary ovarian cancer tissues, as well as healthy human tissues, to detect potential off-tumor staining profiles (exemplary, two binders with different on-/off-target reactivity are shown in Figure 6D, top and middle rows). We quantified signal intensities on single-cell level. Signals of individual scFv-Fcs were compared to the positive reference (Supplementary Table S1A,B). Subsequent analysis of single cells for co-staining with the candidate binder and the positive reference allowed for the identification of FOLR1-specific binder candidates (Figure 6E, top row).

In a second step, binding of scFv-Fc fusion proteins was analyzed on tumor tissue composed of FOLR1-positive and -negative cells (exemplary, shown for two binders on primary ovarian cancer cells in Figure 6D, bottom row). The epithelial cell adhesion molecule (EpCAM) was used as a marker for epithelial tumor cells [46]. Staining patterns of anti-FOLR1 and anti-EpCAM antibodies revealed distinct co-expression patterns, confirming previous findings ([36]; Supplementary Figure S5C). Twelve anti-FOLR1 candidates bound to tumor cells (Figure 6E, bottom row, Supplementary Table S1C). For further selection of scFv-Fc candidates, specificity thresholds were defined for on-target binding (>50% of Jurkat *FOLR1* KI cells), off-target binding (<10% of on OV-90 *FOLR1* KO cells), and on-tumor binding (>50% on-target binding on ovarian cancer tissue). Applying these selection criteria, six candidates were defined as specific for FOLR1 (Figure 6F).

In order to analyze off-tumor binding, we assessed a tissue micro array recommended by the FDA for tissue cross-reactivity studies. Under healthy conditions, FOLR1 expression was described to be restricted to a few organs, including breast, kidney, and lung [47]. Off-tumor binding profiles were determined according to the earlier assessments.

With the exception of the breast and lung, FOLR1 expression was not detected using the FOLR1 positive reference nor any of the anti-FOLR1 scFv-Fc fusion proteins. As previously shown, FOLR1 expression in both tissues was found in distinct anatomical structures and cell types [47].

**Figure 7:**
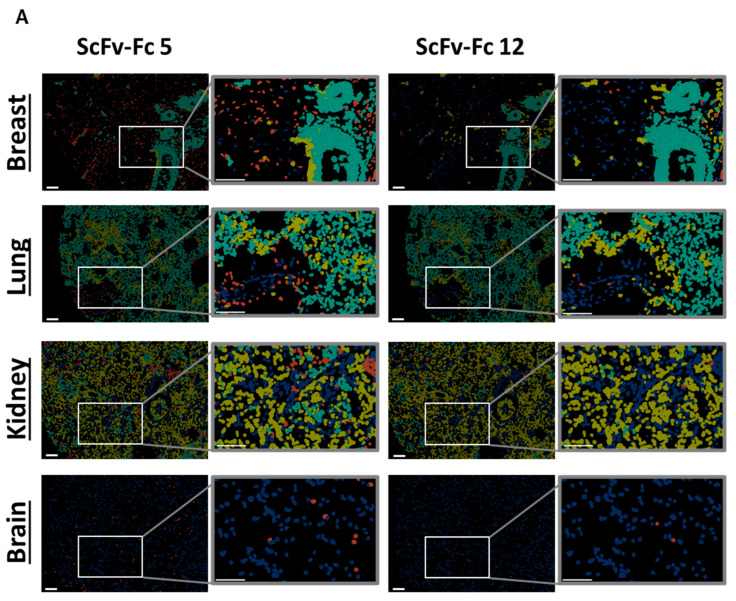

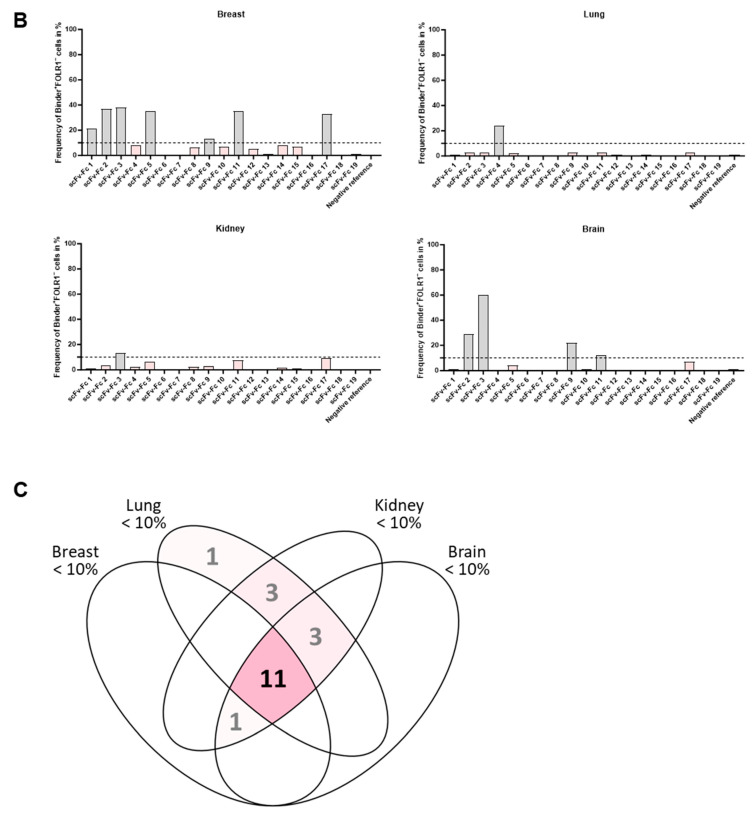
Tissue cross-reactivity workflow identifies anti-FOLR1 binder candidates’ off-tumor binding profile. (**A**) Exemplary representation of selected healthy tissues (breast, lung, kidney, brain) from tissue micro array analysis employing anti-FOLR1 scFv-Fc candidates 5 and 12. Cell segmentation workflow and gating for on-/off-target binding analysis was applied according to Figure 6B. (**B**) Quantification of the off-tumor binding frequency of all binder candidates on breast, lung, kidney, and brain tissue, with total numbers of analyzed cells 1576, 6202, 7494, and 1324, accordingly. Thresholds (dotted line) for evaluation of binder candidate specificity was set to <10% off-tumor binding. (**C**) Venn diagram summarizes off-tumor reactivity on healthy tissues (<10% staining frequency of cells) of new binder candidates. Taken together, 11 candidates match the requirements. Scale bar represents 100 µm.

Variability in on- and off-target binding was observed among candidates on healthy tissues (exemplary shown for two binders with similar on-target but different off-target binding pattern, Figure 7A; all candidates Supplementary Figure S6A–C). Anti-FOLR1 scFv-Fc candidates detected FOLR1-expressing cells in healthy tissues, confirming previously reported FOLR1 expression data [36,47,48]. In order to select specific candidates for further development a cut-off frequency of <10% off-target/off-tumor reactivity was set (Figure 7B, Supplementary Table S1D–F). Applying this selection criteria, 11 candidates were defined as specific (Figure 7C).

By selecting for high specificity, i.e., low off-target binding and reduced on-target off-tumor binding, we identified three candidates (anti-FOLR1 scFv-Fc 12, 14, and 19) with a staining profile similar to the established anti-FOLR1 positive reference antibody. Noteworthy, anti-FOLR1 scFv-Fc candidate 12 revealed the most favorable staining profile, i.e., high frequency of co-staining with the positive reference, as well as low staining frequency of non-target cells. At the same time, this binder showed superior CAR functionality compared to other CAR candidates (summarized in Supplementary Table S2). Consequently, we confirmed the previous decision (based on functional data) of anti-FOLR1 scFv 12 as lead candidate for further development.

## 4. Discussion

Cellular immunotherapy has provided substantial clinical benefits to many patients. Particularly, the use of CAR T cells for the treatment of hematological cancers, but also autoimmune diseases, has improved patient survival significantly [49,50,51]. However, several aspects in the generation and characterization of a CAR can be optimized to further improve the clinical benefit.

We initially hypothesized that several limitations of current CAR T cell approaches, e.g., immunogenicity and off-tumor reactivity, may be addressed early in the development process by comprehensive and complementary assays. As a proof of concept in this study, we set up a new workflow for the identification and selection of CAR candidates based on functionality and on-target/off-tumor reactivity.

Here, we report, (i) the generation of highly specific, fully human scFv sequences from a naïve human B cell library against FOLR1, (ii) the functional high-throughput in vitro assessment of anti-FOLR1 CAR T cells based on these fully human scFv sequences, and (iii) the tissue cross-reactivity characterization of the anti-FOLR1 scFvs to assess off-target and on-target/off-tumor binding.

A fully human CAR design, including extra- and intracellular domains, may decrease the risk of immunogenicity and may consequently improve CAR T cell therapies. The origin and composition of the CAR sequence used, e.g., murine sequences, may induce an immune response in patients after CAR T cell infusion. This anti-CAR immune response may hamper efficacy as well as persistence of CAR T cell treatment [17,18]. Therefore, sequences of murine origin are frequently humanized to mitigate the risk of immunogenicity. Alternatively, fully human sequences from human-derived sources almost completely remove the risk of cellular and/or humoral immunogenicity [21,52,53]. In addition to individual studies reporting human anti-mouse immune reactions, the potential issue of immunogenicity may be even more relevant when CAR T cells are injected multiple times or when different CAR products are applied to a patient.

Consequently, to retrieve fully human scFv sequences, we employed a newly designed human naïve scFv library from B cells. In addition, panning strategies were designed to select highly specific CAR candidates. To isolate antibody fragments recognizing both mouse and human FOLR1, different panning strategies, using either one or both of the targets in consecutive selection rounds, were evaluated. As expected, most antibody hits were identified when both antigens were combined, but also a decent number of cross-reactive clones was identified from the panning strategy, with only mFOLR1. Potentially, dimerization of the target protein and presence of the Fc domain in the human target protein influenced the selection of binders. In summary, alternating murine and human target protein in a phage display selection led to the identification of a high number of cross-reactive antibody fragments.

Additionally, we performed extensive flow cytometric assays to characterize scFv specificity against human and murine FOLR variants. This step addressed scFv binding to its cognate antigen in a physiological context, i.e., full length protein with potential post-translational modifications, without sample fixation prior to assessment. To analyze proper target specificity in a cellular context, we evaluated target recognition on transduced cell lines expressing human and murine FOLR variants. Previous work has shown that CAR functionality is impacted by receptor affinity, whereby both positive and negative effects of affinity tuning have been seen for CAR T cell functionality [54,55]. Therefore, binder selection was not narrowly restricted to high staining frequencies on target cells, but a broad range of scFv-Fc candidates was selected for further evaluation in the CAR format. To select highly specific CAR candidates, they were not only positively selected for hFOLR1 and mFOLR1, but the CAR candidates were also negatively selected against human and murine FOLR2 and FOLR4 variants, respectively. This selection for human and murine FOLR1-specific CAR candidates was critical since the members of the folate receptor family have a high degree of similarity [35].

In contrast, the other reported fully human anti-FOLR1 scFv, C4, was generated using a guided selection approach [56]. In this study, a human Fab fragment was isolated from a phage library using the light chain of the murine monoclonal antibody Mov19 as a template for pairing of human heavy or light chains, respectively, displayed on phages and an ovarian cell line overexpressing FOLR1. Whether the C4 Fab recognizes other FOLR1 variants in human and mouse is unclear.

For functional assessment of CAR molecules, we decided on a second-generation CAR architecture which comprises a CD8a spacer, CD8a transmembrane domain, and a 4-1BB co-stimulatory endodomain (signal 2 for T cell activation), followed by a CD3z-coding endodomain, to provide activation signal 1. We selected 4-1BB as a co-stimulatory domain since it has previously been described to promote proliferation and persistence of T cells [57,58,59]. To assess the impact of individual scFv domains on CAR-mediated target cell lysis, cytokine release, and cell expansion, we compared all CAR constructs in parallel in repeated co-cultures in an automated screening assay. Co-culture assays of CAR T cells, with repetitive addition of target cells, were performed to identify CAR candidates with the best performance in vitro. It was proposed that multiple engagements into T cell effector function, upon repeated antigen exposure, may serve as an in vitro surrogate for the challenging situation CAR T cells may encounter in patients [11,12,13,60]. Although the majority of anti-FOLR1 CAR candidates (17 out of 19) mediated killing of FOLR1-expressing target cells in the first round of co-culture, not all of these anti-FOLR1 CAR candidates (only 12) were able to induce target cell killing in the second and third round. One of these anti-FOLR1 CAR candidates also induced repeated killing of *FOLR1* KO target cells, emphasizing the importance of assessing antigen-independent activation early in the development process. Moreover, this indicates that specificity of a CAR T cell may differ from the specificity of an isolated scFv. This effect may be meditated by different avidities [61]. While most anti-FOLR1 CAR candidates (10 from 12) were able to induce profound release of IFN-γ upon target cell encounter in the third round, only a few anti-FOLR1 CAR candidates (4 out of 12) expanded in all rounds and all three donors. Induction of CAR T cell proliferation underlined their functional superiority [62]. These findings indicate that CAR T cell functionality changes after repeated contact with target cells, highlighting that advanced in vitro assays are critical for functional assessment of CAR T cells.

Subsequently, we performed additional in vitro assays to further characterize the anti-FOLR1 CAR T cell candidates under challenging conditions, i.e., repetitive killing assays at low effector-to-target cell ratios (1:2), and selected the best performing candidates.

So far, one other fully human anti-FOLR1 CAR, C4-27z CAR, was reported [63]. The reported C4-27z CAR and our described fully human anti-FOLR1 CAR differ in the co-stimulatory domains, where the C4-27z CAR incorporates a CD27 domain and our fully human anti-FOLR1 CAR bears a 4-1BB endodomain. CD27 (also known as TNFRSF7) is a member of the TNFR superfamily, and it is expressed by immune cells [63]. The authors hypothesized that CD27 co-stimulation may enhance expansion, effector function, and survival in vitro. Additionally, CD27 co-stimulation should support human CAR T-cell persistence and anti-tumor activity in vivo [64]. However, a comparison of CD27 against CD28 or 4-1BB co-stimulation was not performed by the authors to show a benefit of CD27 signaling. Although the C4-27z CAR showed promising in vitro and in vivo data, it was not assessed in repetitive killing assays, which is an in vitro dysfunctional model [65]. The fully human anti-FOLR1 CAR which we described was selected based on its functionality in this dysfunctionality model.

In addition to the extensive functional in vitro characterization of CAR T cell candidates, we set up a new tissue cross-reactivity workflow based on multiplexed immunofluorescence. We aimed to include on-target/off-tumor reactivity early in the selection process, since several clinical CAR T cell studies reported either off-target or on-target/off-tumor reactivity of CAR T cells [22]. Examples for on-target/off-tumor reactivity are CAR T cells targeting ErbB2/her2 [29], EGFR [27], and CAIX-specific CAR T cells [66]. By assessment of off-target or on-target/off-tumor reactivity early in the development process, we intended to focus on the most promising candidates and efficiently perform the subsequent development.

For evaluating off-tumor and off-target reactions specifically related to CAR T cell therapy, the FDA recommends several assays that are crucial for evaluating the safety and efficacy of CAR T cell products. Tissue cross-reactivity studies provide information from more complex tissue samples and help to identify potential off-target and off-tumor interactions that may lead to adverse effects in patients.

Spatial multiomics offers several advantages over traditional FDA-recommended assays for assessing off-target reactions for CAR T cells. Spatial multiomics techniques, such as spatial transcriptomics and spatial proteomics, provide detailed spatial information about gene expression, protein expression, and cell–cell interactions within tissue samples. This comprehensive spatial data allow for the identification of off-tumor and off-target interactions within the context of the tissue microenvironment, providing insights into potential off-tumor effects that may not be captured by traditional assays.

The tissue cross-reactivity workflow started with an on-target analysis of the CAR candidates. Cell lines with defined FOLR1 expression, i.e., knock-in or knock-out, respectively, were used to determine on-target reactivity. Here, we already excluded candidates which did not meet our criteria. Next, we analyzed the on-tumor binding profile of the anti-FOLR1 scFv-Fc fusion proteins using primary human ovarian cancer tissues.

It was previously described that FOLR1 expression is not restricted to ovarian cancer but can also be found in healthy breast, kidney, and lung tissue. This indicates off-tumor expression of FOLR1. FOLR1 expression is locally restricted to the apical surface of epithelial cells, which may make the receptor potentially inaccessible to blood-circulating drugs [36,67]. Consequently, analysis of off-tumor binding of new CAR candidates targeting FOLR1 is important.

Subsequent analysis of the anti-FOLR1 scFv-Fc binder candidates on non-malignant tissues enabled further exclusion of candidates with potential off-target specificity. The analysis of tissue micro arrays allowed for the reduction of the number of potential candidates with lowest off-target specificity to three. Finally, we nominated a lead candidate for further development based on superior performance in comprehensive functionality assays as well as most promising safety profile.

In contrast, the other reported fully human anti-FOLR1 scFv, C4, was exclusively tested for on-tumor reactivity on ovarian carcinoma by immunohistochemistry [56].

A limitation of the study is that the scFv-Fc fusion proteins may not fully reflect the interaction of a CAR T cell with its cognate antigen. The interaction of a CAR T cell with an antigen-expressing cell may be modulated by multiple interactions, giving rise to an avidity that cannot be resembled by a single molecule like a scFv-Fc.

Albeit, the FOLR1 expression in mice has not fully been investigated yet [68], mouse–human cross-reactive binders—as characterized in this study—could be a valuable tool to address on-target, off-tumor expression of FOLR1 in murine tissue and potential on-target, off-tumor related toxicity in mice. Future studies should address whether FOLR1 expression in mice is comparable to human FOLR1 expression in terms of tissue distribution, cellular localization, and intensity. Subsequently, it could be assessed if cross-reactive scFvs bind to murine tissues expressing FOLR1 to foresee potential toxicities. Finally, such a hypothesis could be confirmed by applying murine-reactive CAR T cells to mice and carefully monitor animals for potential toxicities.

Besides a safety assessment in mice, future studies should address efficacy of the fully human anti-FOLR1 CAR in a relevant in vivo ovarian cancer model. These data will support the clinical translation of a fully human anti-FOLR1 CAR T cell therapy for a broad range of FOLR1-expressing epithelial cancers.

Taken together, we describe a novel workflow to identify and characterize fully human lead CAR candidates. Our approach combines a series of complementary assays including highly specific candidate selection from a human antibody phage library with positive and negative selection steps. Moreover, lead candidate selection was based on CAR functionality as well as on-target/off-tumor reactivity to mitigate potential toxicities. Thereby, CAR therapy may be improved by more efficient lead selection and reduced risk of potential toxicities in patients.

## 5. Conclusions

This study presents a novel workflow for generating fully human CAR T cells by integrating comprehensive assays to address current limitations of CAR development, such as immunogenicity and off-tumor reactivity. By selecting CAR candidates with superior functionality and minimal off-target effects, our approach shows promise for improving the safety and efficacy of CAR T cell therapies. Future studies will be needed to confirm these findings in relevant in vivo models and further support their clinical translation.

## Data Availability

All data generated or analyzed during this study are included in this article. Further inquiries can be directed to the corresponding author upon reasonable request.

## Supplementary Materials

The following supporting information can be downloaded at: https://www.mdpi.com/article/10.3390/cells13221880/s1, Figure S1: ELISA screening of selected scFvs, Figure S2: Flow cytometric analysis enables identification of FOLR1-specific scFv-Fc candidates, as well as exclusion of off-target binders, Figure S3: In vitro CAR T cell screening identifies CAR T cell candidates endowed with off-target cytotoxicity, Figure S4: Advanced in vitro CAR T cell assays confirm functional lead anti-FOLR1 CAR T cell candidate from additional donors on alternative ovarian cancer cell lines, Figure S5: Cyclic imaging of staining with FOLR1-specific binders allows for their comparison and identification of the lead candidates with high on-target and low off-target specificity, Figure S6: Cyclic imaging of staining with FOLR1-specific binders allows for their comparison and identification of the lead candidates with high on-target and low off-target specificity, Table S1: Quantification of the detected signal from all binder candidates, their correlation to the positive and negative references enables identification of the best binder candidates with high on-target and low off-target specificities, Table S2: Summary of the identification and selection workflow assays for novel, fully human CAR T cell lead candidates, Table S3: Reagent list.
