# Supplementary material for: Identification and Characterization of Fully Human FOLR1-Targeting CAR T Cells for the Treatment of Ovarian Cancer
Source: Cells. 2024 Nov 14;13(22):1880. doi: 10.3390/cells13221880 (PMC11592683; doi:10.3390/cells13221880)
Supplement: Supplementary file 1 [file cells-13-01880-s001.zip › cells-3256839-supplementary.pdf]

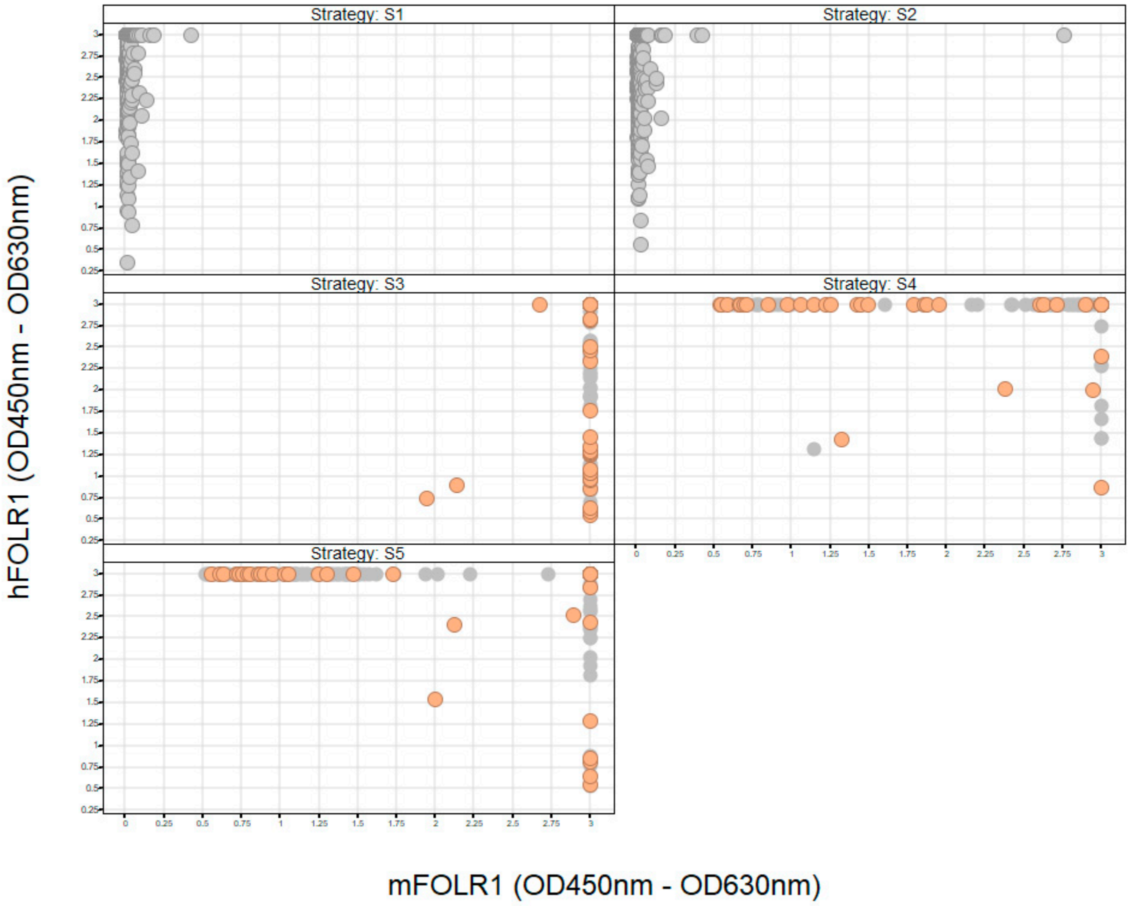

Supplementary Figure S1. ELISA screening of selected scFvs. After panning of a phage display antibody library 2,266 first hits for hFOLR1 and/or mFOLR1 were identified in ELISA. The five different selection strategies lead to the discovery of scFvs that bind preferably to the target(s) that were used in the panning strategies, e.g. panning strategies 4 and 5 selected scFvs were binding preferably to both hFOLR1 and mFOLR1 antigen. The 189 selected candidates chosen for validation by flow cytometry are highlighted in orange. Non-selected candidates as well as sequence identical candidates are shown in grey.

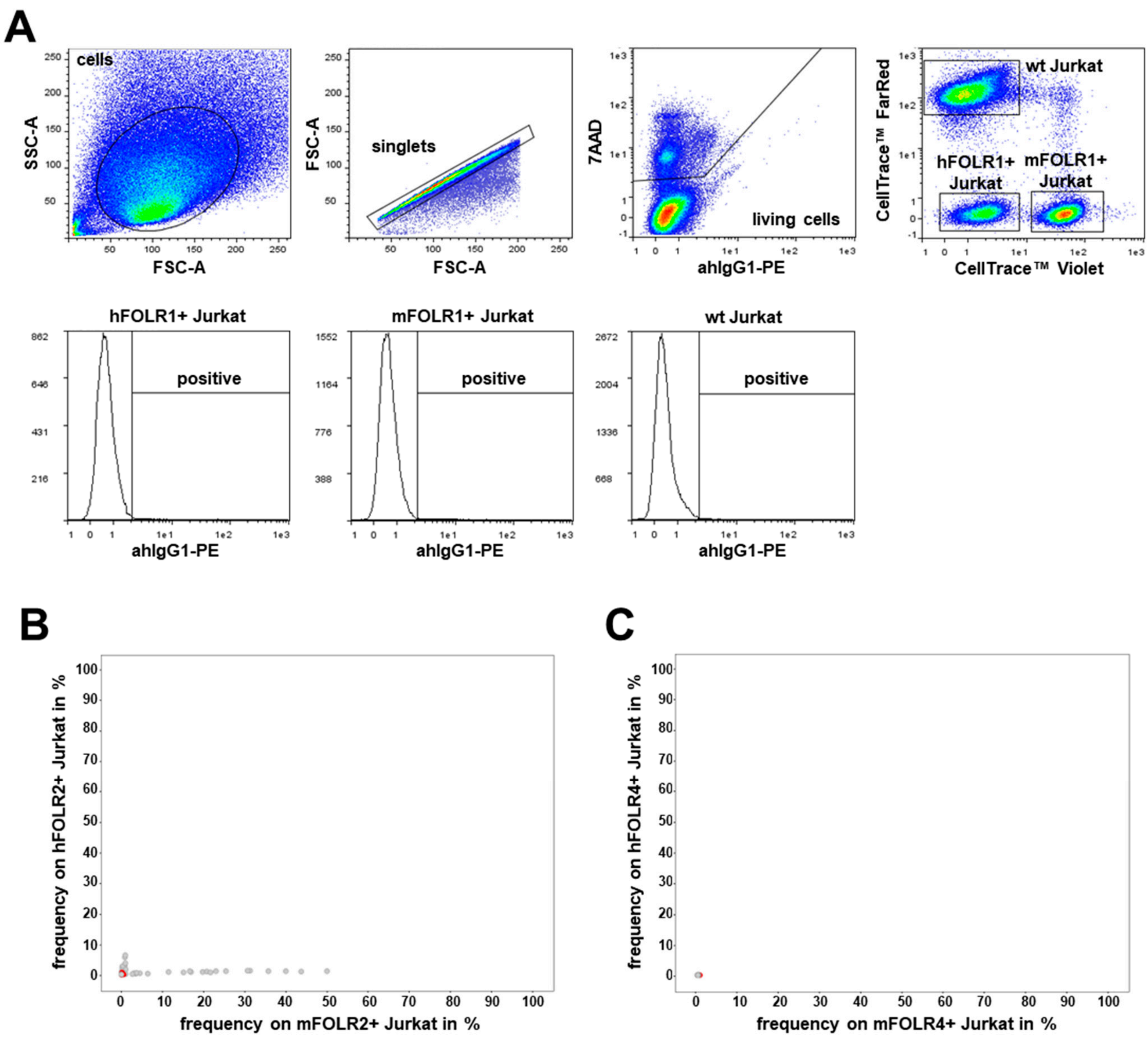

Supplementary Figure S2. Flow cytometric analysis enables identification of FOLR1 specific scFv-Fc candidates as well as exclusion of off-target binders. (**A**) Gating strategy for the screening of anti-FOLR1 scFv-Fc on Jurkat cells expressing different FOLR variants. Jurkat cells were gated on size, singularity and viability (7AAD-) before distinguishing between hFOLR1, mFOLR1, and wt Jurkat cells by CellTracer staining. Specificity of the respective scFv-Fc candidate was identified by ahIgG1-PE staining among the target and non-target cell lines. To exclude off-target binders that recognize (**B**) hFOLR2, mFOLR2, (**C**) hFOLR4, or mFOLR4 flow cytometric staining on Jurkat cells transduced with the respective FOLR variant, was performed. Graphs represent data from three independent experiments and show data as mean values. Lead candidates are marked in red. Binders that do not meet the selection criteria are marked in grey. Thresholds frequency on off-target cell line < 1%.

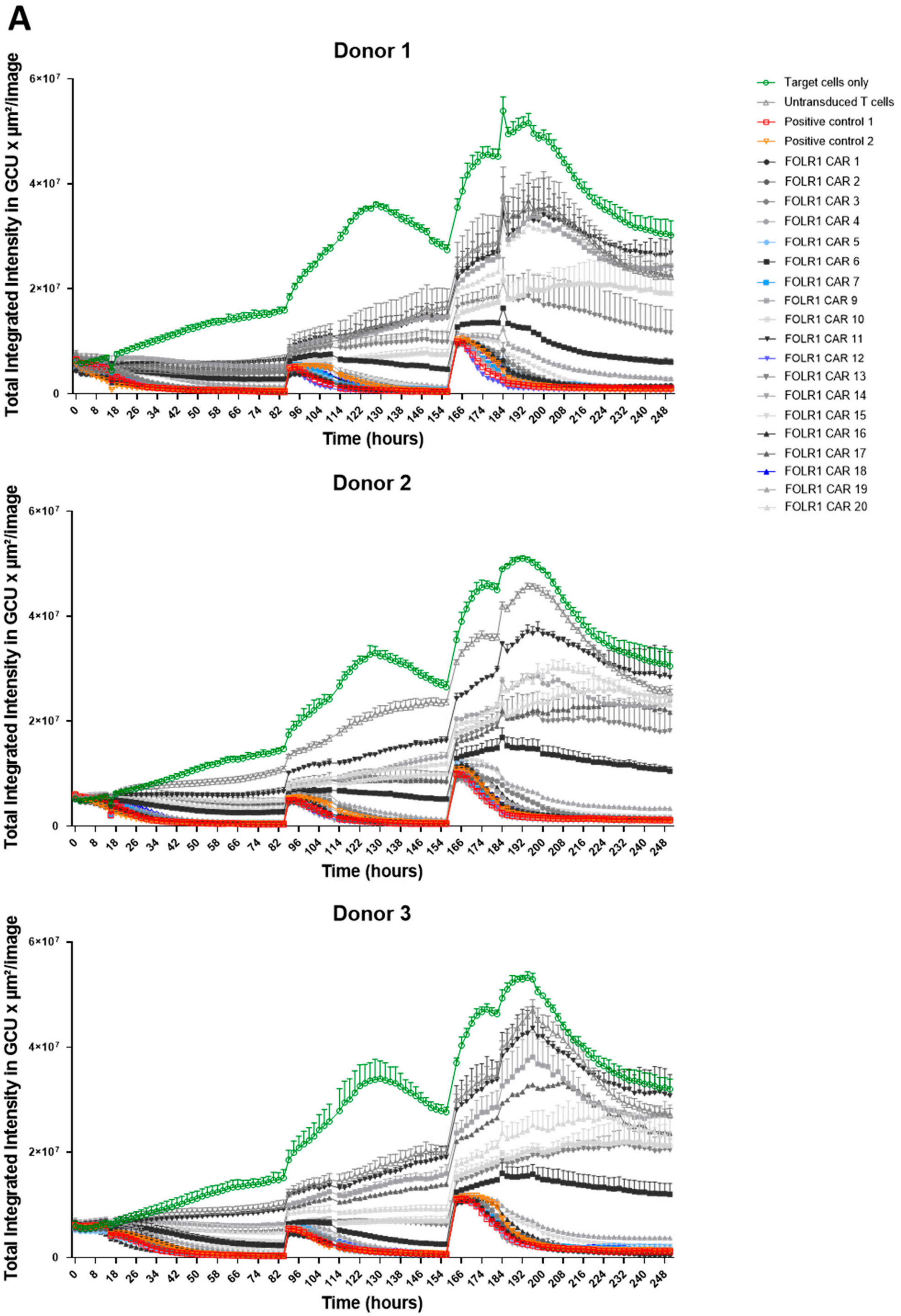
A
Donor 1
Donor 2
Donor 3
Total Integrated Intensity in GCU x µm²/image
Time (hours)
Target cells only
Untransduced T cells
Positive control 1
Positive control 2
FOLR1 CAR 1
FOLR1 CAR 2
FOLR1 CAR 3
FOLR1 CAR 4
FOLR1 CAR 5
FOLR1 CAR 6
FOLR1 CAR 7
FOLR1 CAR 9
FOLR1 CAR 10
FOLR1 CAR 11
FOLR1 CAR 12
FOLR1 CAR 13
FOLR1 CAR 14
FOLR1 CAR 15
FOLR1 CAR 16
FOLR1 CAR 17
FOLR1 CAR 18
FOLR1 CAR 19
FOLR1 CAR 20

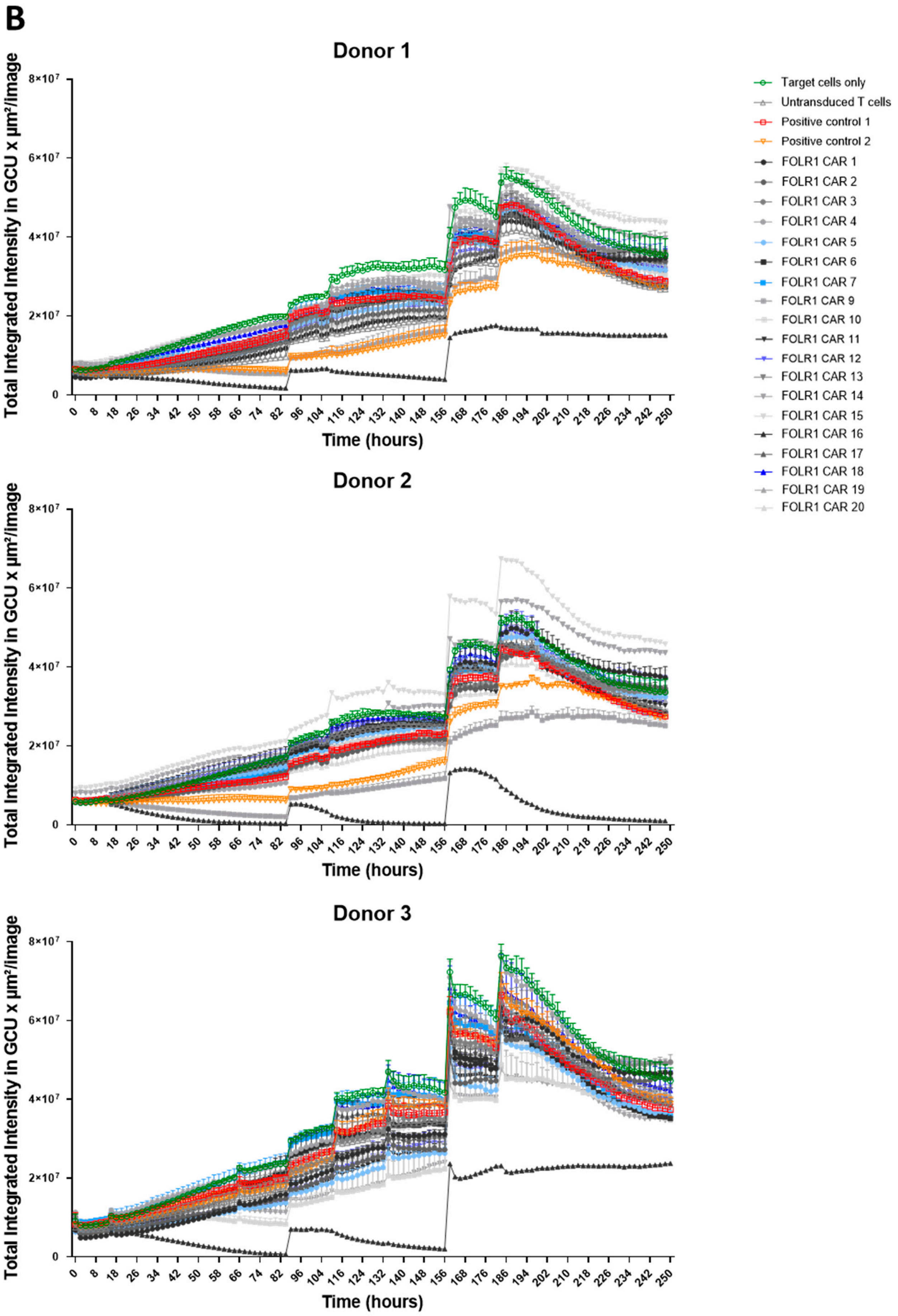
B
Donor 1
Donor 2
Donor 3
Total Integrated Intensity in GCU x µm²/image
Time (hours)
Target cells only
Untransduced T cells
Positive control 1
Positive control 2
FOLR1 CAR 1
FOLR1 CAR 2
FOLR1 CAR 3
FOLR1 CAR 4
FOLR1 CAR 5
FOLR1 CAR 6
FOLR1 CAR 7
FOLR1 CAR 9
FOLR1 CAR 10
FOLR1 CAR 11
FOLR1 CAR 12
FOLR1 CAR 13
FOLR1 CAR 14
FOLR1 CAR 15
FOLR1 CAR 16
FOLR1 CAR 17
FOLR1 CAR 18
FOLR1 CAR 19
FOLR1 CAR 20

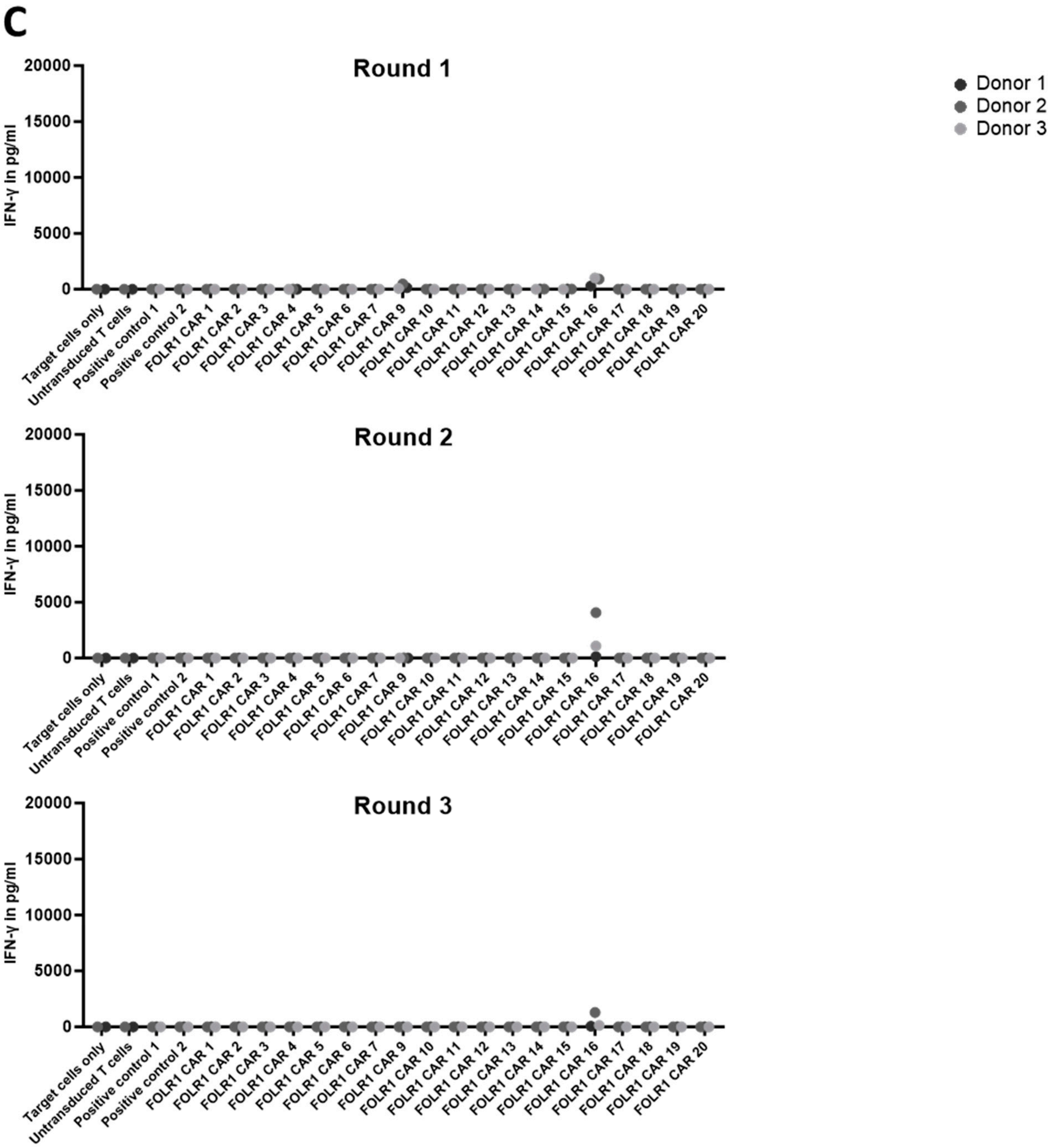

Supplementary Figure S3. *In vitro* CAR T cell screening identifies CAR T cell candidates endowed with off-target cytotoxicity. (**A**) Ability to induce lysis of ovarian cancer cells proficient for FOLR1 (OV-90) by all CAR T cell candidates of three different donors. Error bars indicate respective mean value and SD. (**B**) To measure antigen-independent lysis of ovarian cancer cells GFP-expressing OV-90 *FOLR1* KO were seeded and co-cultured with CAR T cells. After 92 h of co-culture, fresh OV-90 *FOLR1* KO cells were added in addition, followed by a second addition of OV-90 *FOLR1* KO cells after 164 h. Target cell lysis by the CAR T cell candidates was analyzed by decrease in GFP signal over time by measuring the Green Calibrated Units per μm²/image for 11 days. As controls, target cells (OV-90 *FOLR1* KO) were cultured without addition of CAR T cells or co-cultured with untransduced T cells. Two positive control CAR constructs known to successfully induce FOLR1-specific lysis of target cells by CAR T cells were included. Error bars indicate SD of respective mean value. (**C**) IFN-γ secretion in repeated co-cultures 24 h after each addition of ovarian cancer cells deficient for FOLR1 (OV-90 *FOLR1* KO) to candidate CAR T cells.

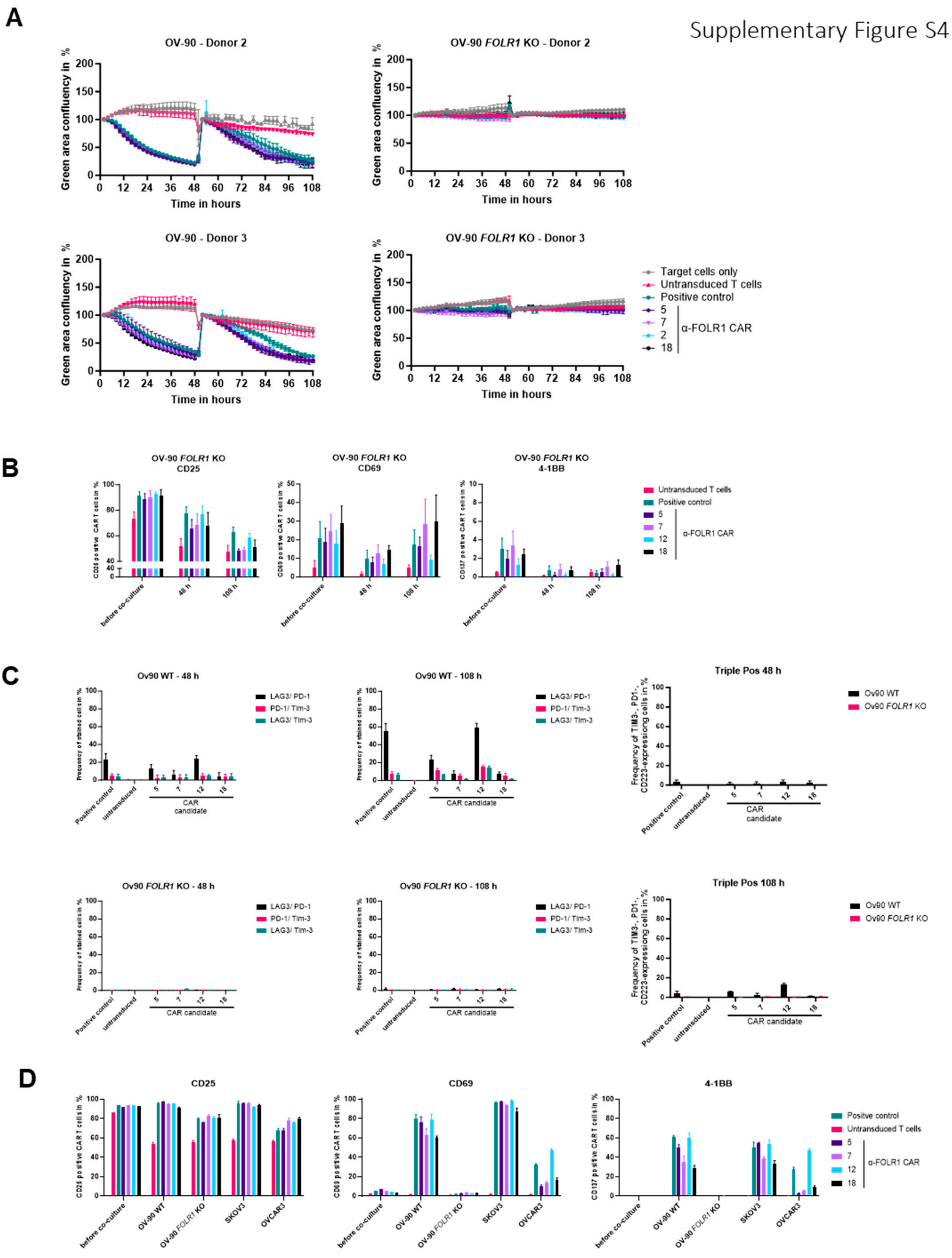
Supplementary Figure S4
A
OV-90 - Donor 2
OV-90 FOLR1 KO - Donor 2
OV-90 - Donor 3
OV-90 FOLR1 KO - Donor 3
Green area confluency in %
Time in hours
Target cells only
Untransduced T cells
Positive control
5
7
2
18
α-FOLR1 CAR
B
OV-90 FOLR1 KO CD25
OV-90 FOLR1 KO CD69
OV-90 FOLR1 KO 4-1BB
before co-culture
48 h
108 h
Untransduced T cells
Positive control
5
7
12
18
α-FOLR1 CAR
C
Ov90 WT - 48 h
Ov90 WT - 108 h
Triple Pos 48 h
Ov90 FOLR1 KO - 48 h
Ov90 FOLR1 KO - 108 h
Triple Pos 108 h
LAG3/ PD-1
PD-1/ Tim-3
LAG3/ Tim-3
Frequency of stained cells in %
Frequency of TIM3-, PD1-, CD223-expressiong cells in %
Ov90 WT
Ov90 FOLR1 KO
Positive control
untransduced
CAR candidate
D
CD25
CD69
4-1BB
before co-culture
OV-90 WT
OV-90 FOLR1 KO
SKOV3
OVCAR3
Positve control
Untransduced T cells
5
7
12
18
α-FOLR1 CAR

Supplementary Figure S4. Advanced *in vitro* CAR T cell assays confirm functional lead anti-FOLR1 CAR T cell candidate from additional donors on alternative ovarian cancer cell lines. (**A**) Killing assay of four CAR T candidates from additional donors co-cultured with FOLR1-proficient GFP-expressing ovarian cancer cells expressing (OV-90, left) and FOLR1-deficient GFP-expressing ovarian cancer cells expressing (OV-90 *FOLR1* KO, right). Data points represent mean values and SD is indicated. (**B**) Expression of activation markers CD25, CD69, and 4-1BB was analyzed at indicated time points by flow cytometry (three donors) on OV-90 *FOLR1* KO. (**C**) Double or triple expression of exhaustion markers LAG3, PD1, and TIM3 at the indicated time points was analyzed by flow cytometry (three donors) on OV-90 and OV-90 *FOLR1* KO. Bars represent mean values and SD is depicted. (**D**) Expression of activation markers CD25, CD69, and 4-1BB was analyzed after 48 h by flow cytometry (single donor) on ovarian cancer cell lines OV-90, OV-90 *FOLR1* KO, SKOV3, and OvCAR3, respectively.

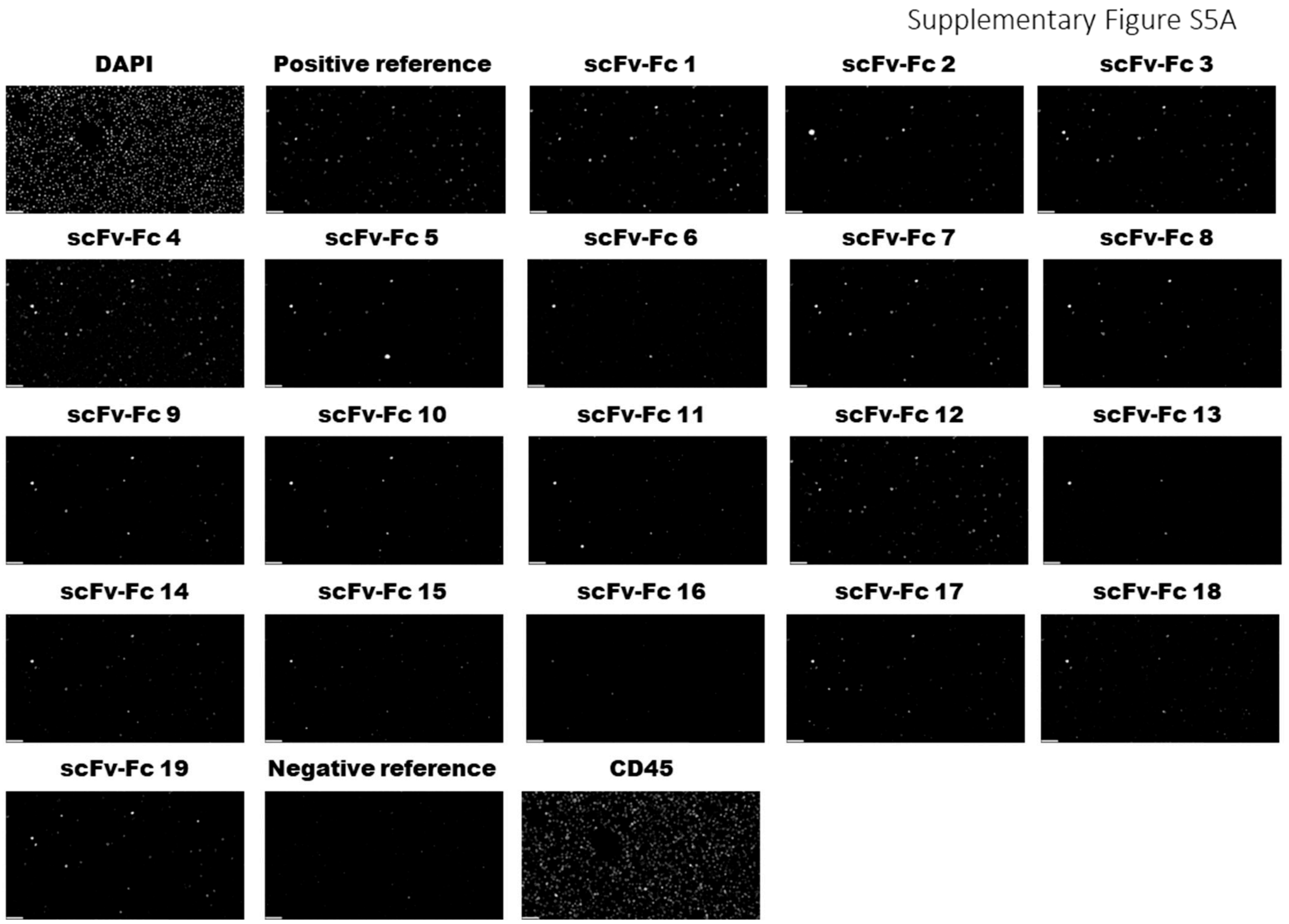
Supplementary Figure S5A
DAPI
Positive reference
scFv-Fc 1
scFv-Fc 2
scFv-Fc 3
scFv-Fc 4
scFv-Fc 5
scFv-Fc 6
scFv-Fc 7
scFv-Fc 8
scFv-Fc 9
scFv-Fc 10
scFv-Fc 11
scFv-Fc 12
scFv-Fc 13
scFv-Fc 14
scFv-Fc 15
scFv-Fc 16
scFv-Fc 17
scFv-Fc 18
scFv-Fc 19
Negative reference
CD45

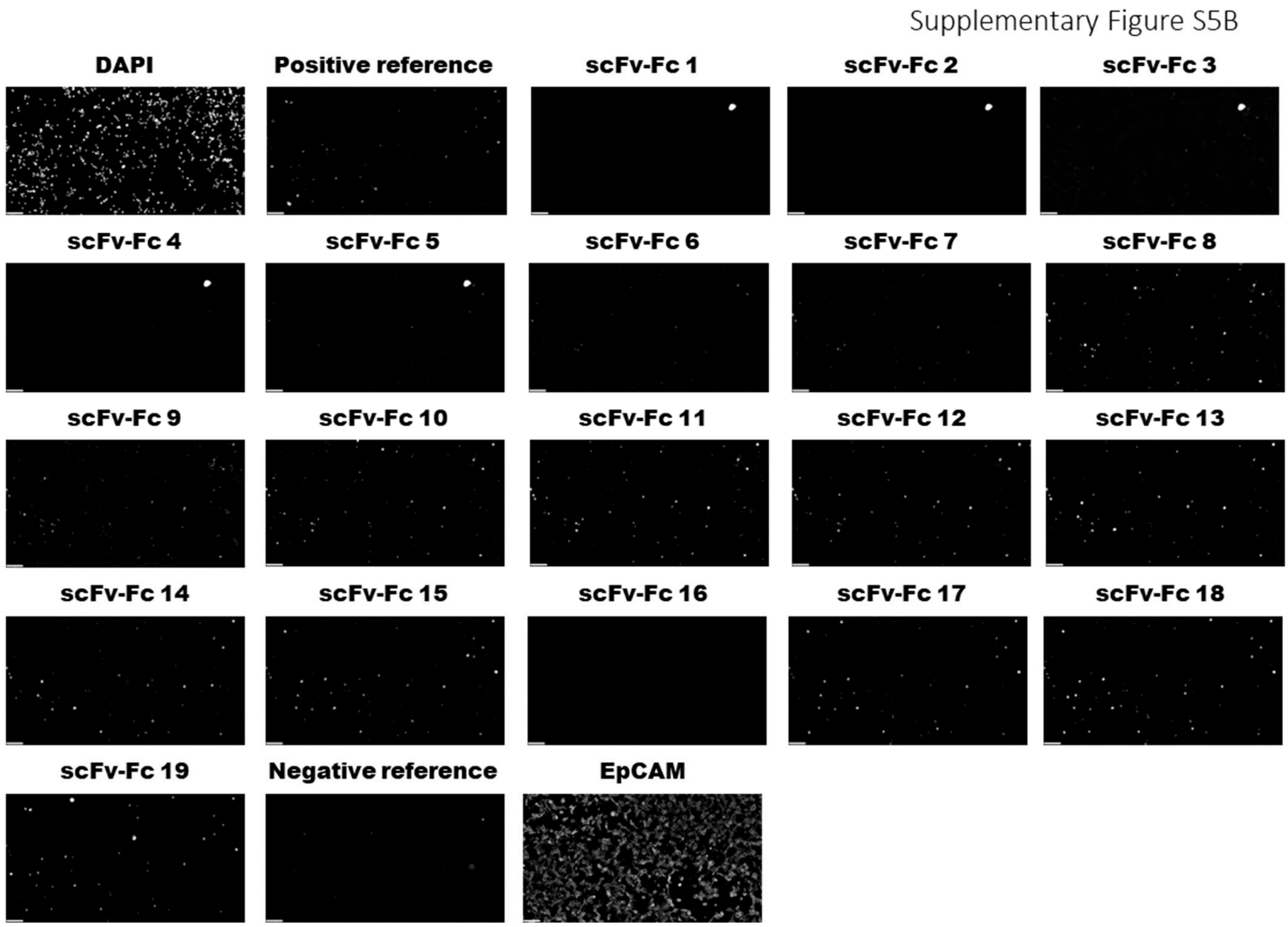
Supplementary Figure S5B
DAPI
Positive reference
scFv-Fc 1
scFv-Fc 2
scFv-Fc 3
scFv-Fc 4
scFv-Fc 5
scFv-Fc 6
scFv-Fc 7
scFv-Fc 8
scFv-Fc 9
scFv-Fc 10
scFv-Fc 11
scFv-Fc 12
scFv-Fc 13
scFv-Fc 14
scFv-Fc 15
scFv-Fc 16
scFv-Fc 17
scFv-Fc 18
scFv-Fc 19
Negative reference
EpCAM

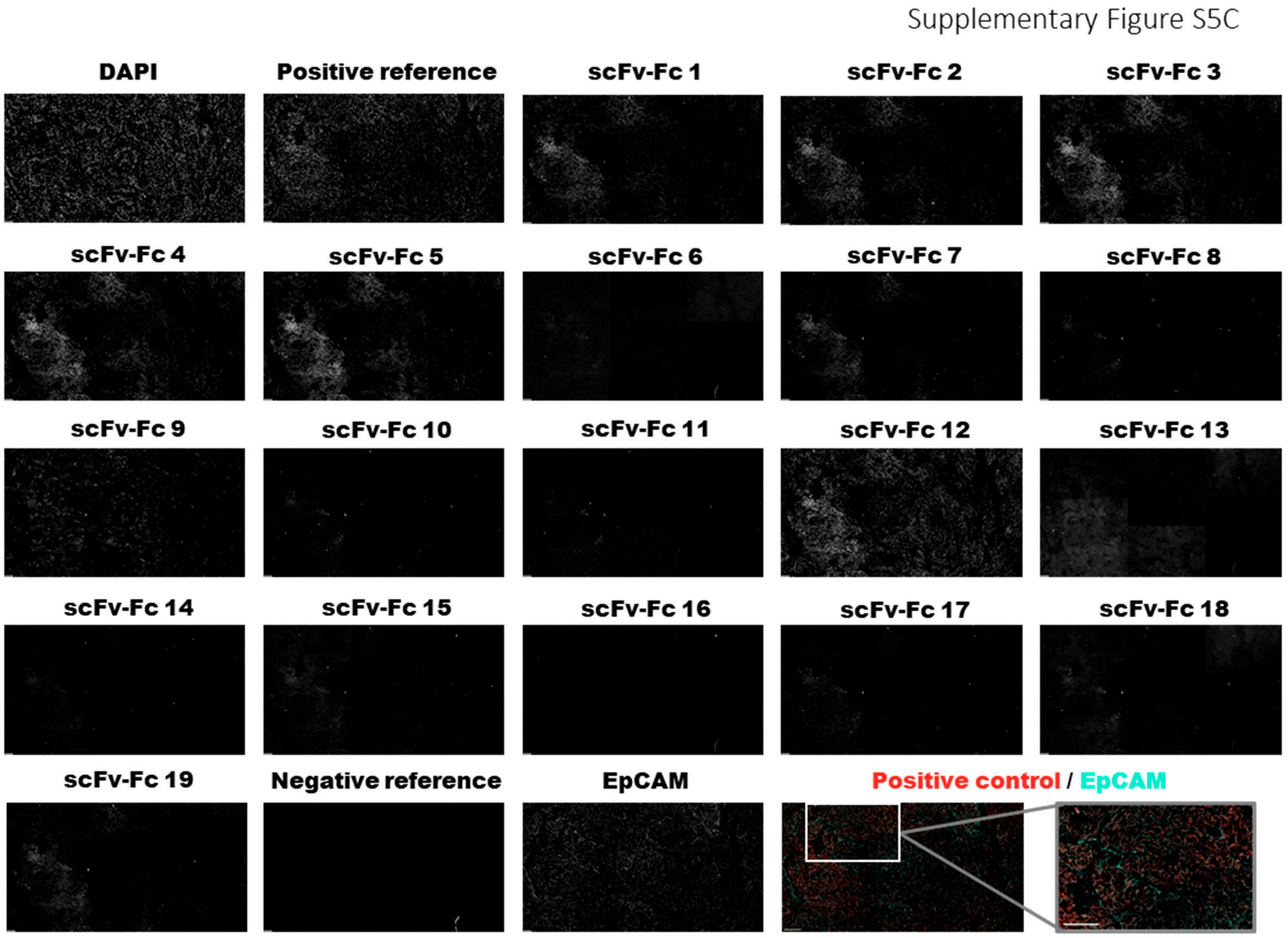

Supplementary Figure S5. Cyclic imaging of staining with FOLR1-specific binders allows for their comparison and identification of the lead candidates with high on-target and low off-target specificity. (**A-F**) Staining patterns of all scFv-Fc candidates, the positive reference (anti-FOLR1 monoclonal antibody LK26) and the negative reference on the cell lines, and malignant tissue. (**A**) Staining on Jurkat *FOLR1* KI cell line with transgenic expression of human FOLR1. DAPI and CD90 staining were added as controls for detection of all Jurkat cells. (**B**) Staining on OV-90 *FOLR1* KO cell line with CRISPR/Cas9-mediated knock-out of human *FOLR1*. DAPI and EpCAM staining were added as controls for detection of all target malignant cells. (**C**) Staining on primary human ovarian cancer tissue.

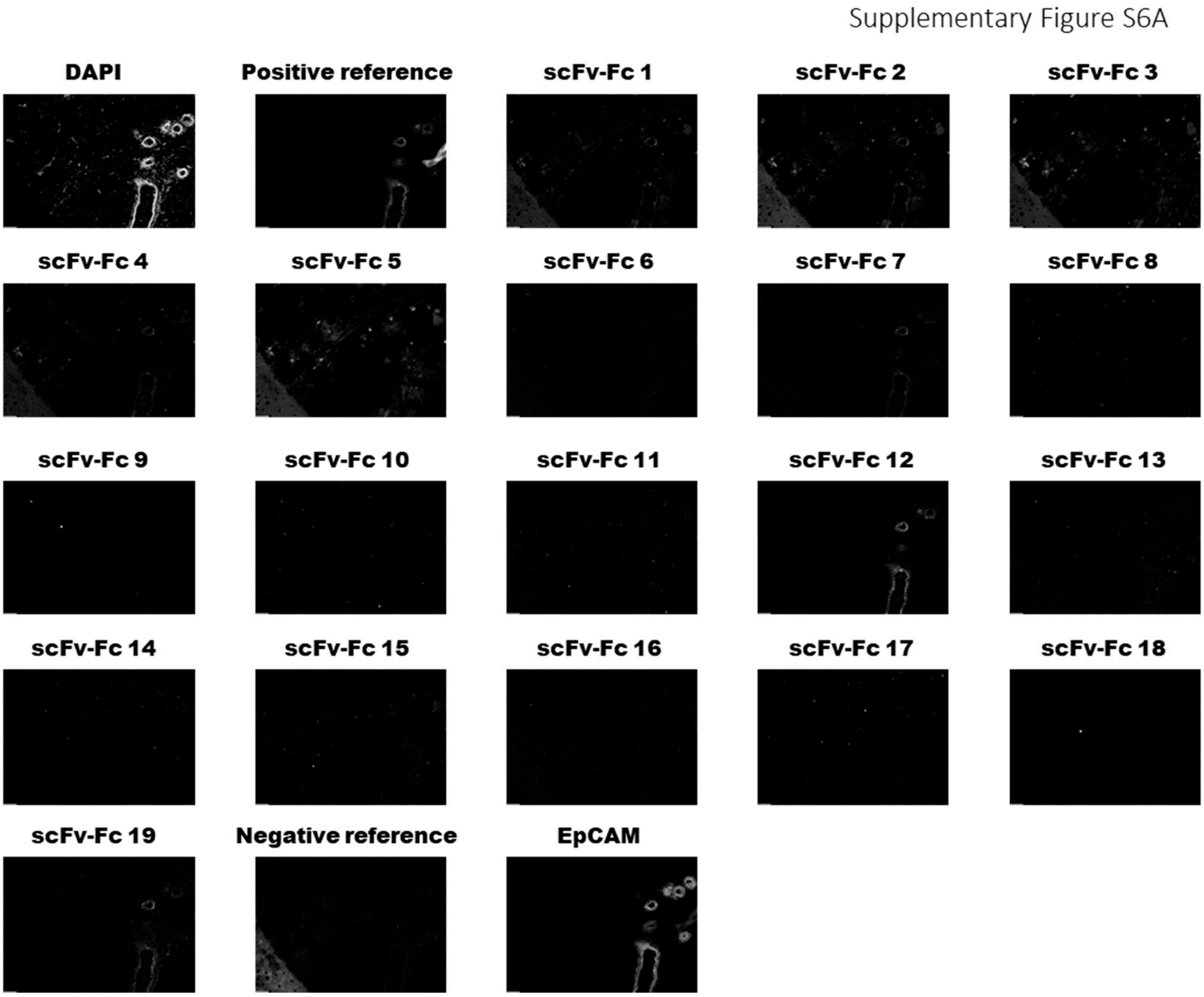
Supplementary Figure S6A
DAPI
Positive reference
scFv-Fc 1
scFv-Fc 2
scFv-Fc 3
scFv-Fc 4
scFv-Fc 5
scFv-Fc 6
scFv-Fc 7
scFv-Fc 8
scFv-Fc 9
scFv-Fc 10
scFv-Fc 11
scFv-Fc 12
scFv-Fc 13
scFv-Fc 14
scFv-Fc 15
scFv-Fc 16
scFv-Fc 17
scFv-Fc 18
scFv-Fc 19
Negative reference
EpCAM

Supplementary Figure S6B

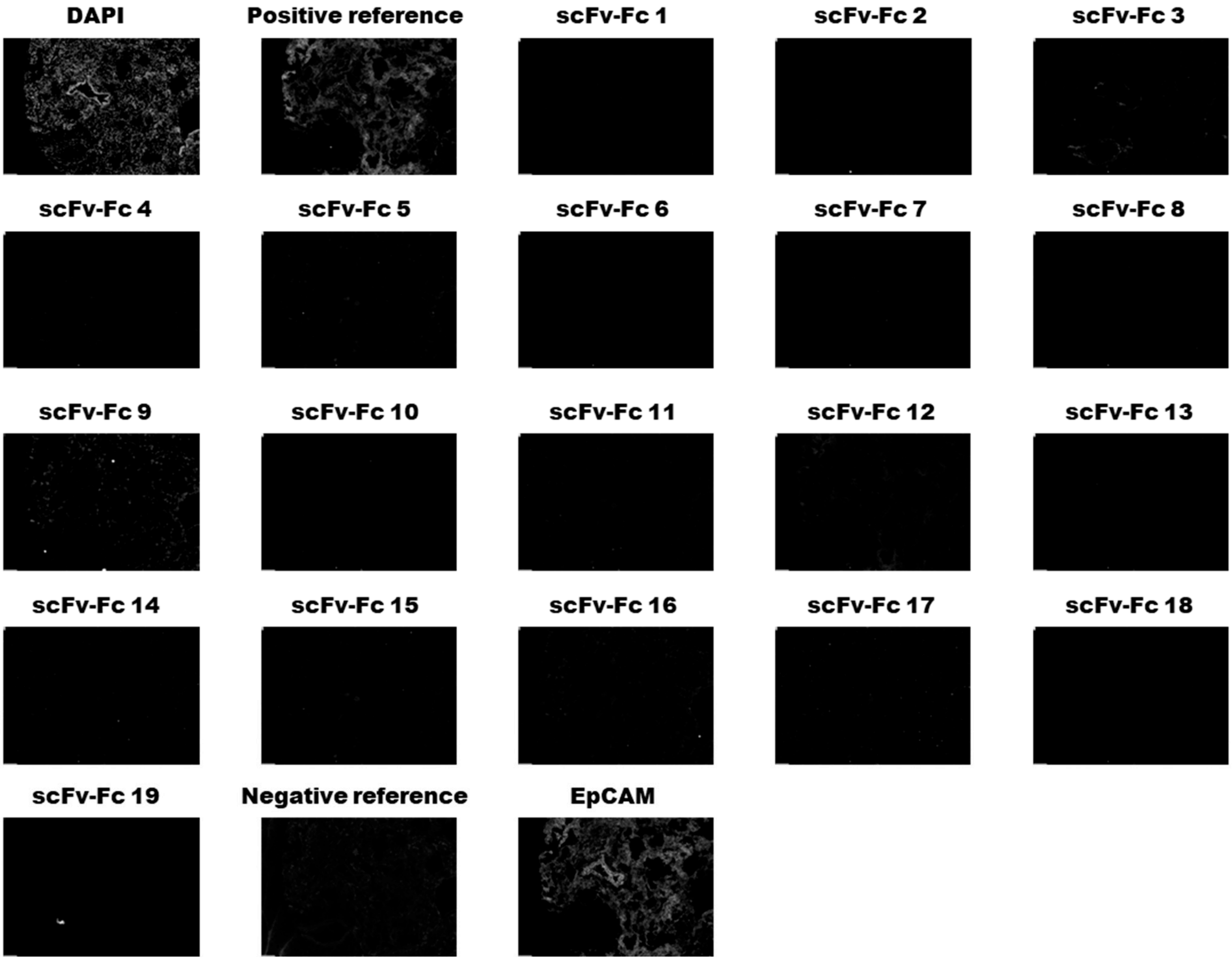

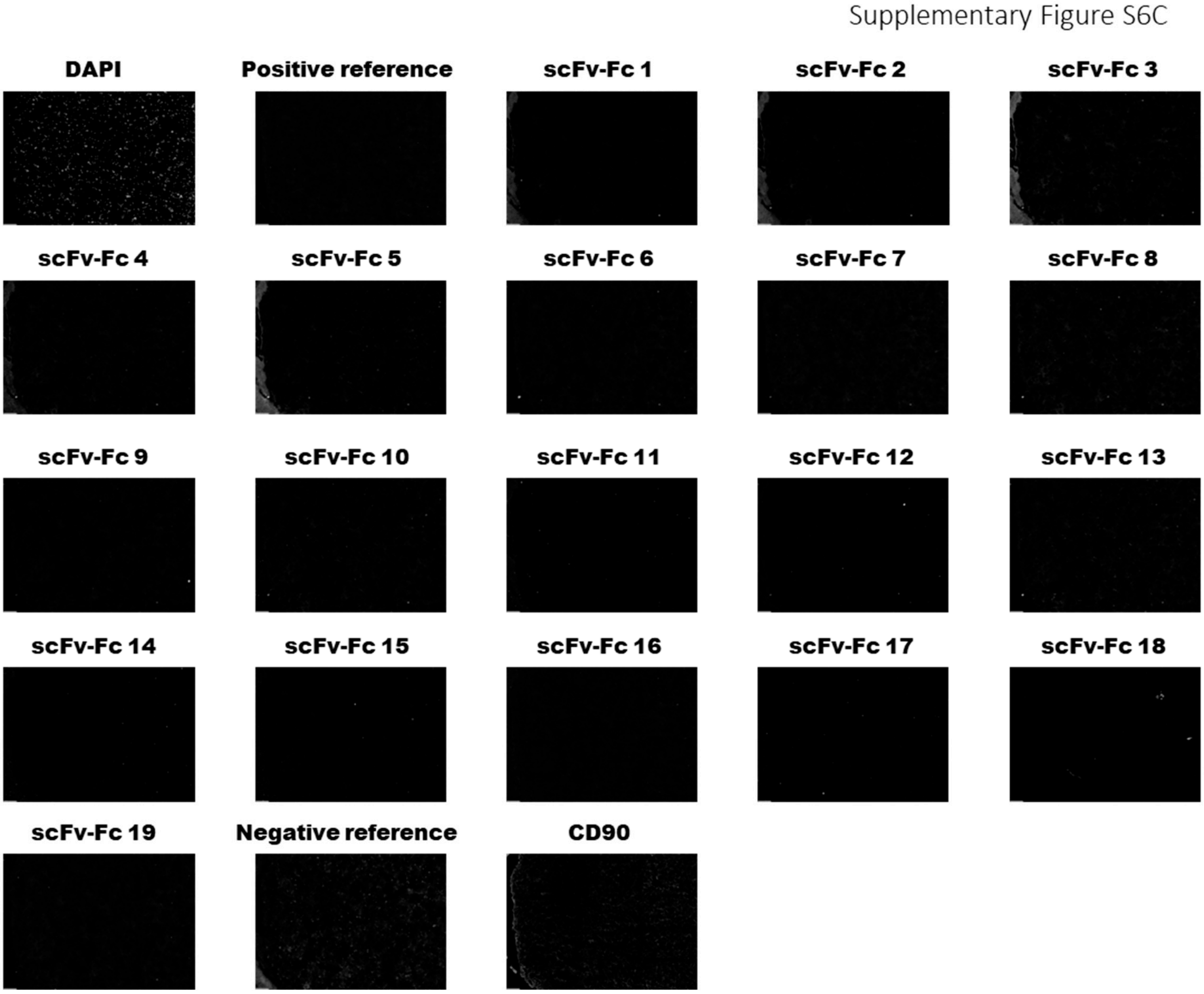

Supplementary Figure S6. Cyclic imaging of staining with FOLR1-specific binders allows for their comparison and identification of the lead candidates with low off-tumor specificity. (**A-C**) Staining patterns of all scFv-Fc candidates, the positive reference (anti-FOLR1 monoclonal antibody LK26) and the negative reference on healthy tissues. (**A**) Staining on tissue micro array from healthy human breast tissue. (**B**) Staining on tissue micro array from healthy human lung tissue. (**C**) Staining on tissue micro array from healthy human brain tissue.

Supplementary Table S1. Quantification of the detected signal from all binder candidates, their correlation to the positive and negative references enables identification of the best binder candidates with high on-target and low off-target specificities. (**A-F**) Calculations were performed by application of the general gating strategy based on the mean intensity of the negative reference as the threshold value for specific signal. The obtained parameters were mean intensity of the detected signals, the correlation of binder signals to the positive reference (relative intensity and frequency of double positive scFv-Fc^{+}/FOLR1^{+} cells), the off-target binding on the non-target FOLR1-negative cells (frequency of scFv-Fc^{+}/FOLR1^{-} cells), as well as co-staining with the negative reference. Quantification of all parameters was carried out upon staining of (**A**) Jurkat *FOLR1* KI cell line, (**B**) OV-90 *FOLR1* KO cell line (**C**) primary human ovarian cancer tissue, (**D**) healthy human breast tissue, (**E**) healthy human lung tissue, (**F**) healthy human brain tissue. N/A: non-applicable, a.u.: arbitrary units.

Supplementary Table S1A

Candidate	Mean intensity, in a.u.	Relative intensity to positive reference, in %	Frequency of scFv-Fc+/FOLR1+, double positive cells in %	Frequency of scFv-Fc+/FOLR1- cells (off-target), in %	Frequency of marker and negative reference, double positive cells in %
scFv-Fc 1	113.5	16.4	81	0.23	1,75
scFv-Fc 2	227.8	32.9	92	0.64	1,80
scFv-Fc 3	71.9	10.4	88	0.40	1,80
scFv-Fc 4	50.2	7.3	80	0.29	1,80
scFv-Fc 5	57.6	8.3	88	0.35	1,80
scFv-Fc 6	20.7	3.0	26	0	0,93
scFv-Fc 7	25.0	3.6	23	0	1,34
scFv-Fc 8	26.1	3.8	33	0.23	1,45
scFv-Fc 9	37.7	5.4	81	0.23	1,75
scFv-Fc 10	36.9	5.3	85	0.29	1,75
scFv-Fc 11	44.2	6.4	85	0.52	1,75
scFv-Fc 12	198.2	28.6	93	0.35	1,80
scFv-Fc 13	22.0	3.2	30	0.11	1,45
scFv-Fc 14	39.7	5.7	69	0.29	1,75
scFv-Fc 15	22.2	3.2	23	0.17	1,40
scFv-Fc 16	12.5	1.8	2	0.05	0,058
scFv-Fc 17	52.7	7.6	87	0.35	1,75
scFv-Fc 18	64.2	9.3	91	0.29	1,80
scFv-Fc 19	39.9	5.8	79	0.11	1,80
FOLR1 (positive reference)	692.3	100	N/A	N/A	1,80
VH36 (negative reference)	13.6	2.0	N/A	N/A	N/A

Supplementary Table S1B

Candidate	Mean intensity, in a.u.	Relative intensity to positive reference, in %	Frequency of scFv-Fc$^+$/FOLR1$^+$, double positive cells in %	Frequency of scFv-Fc$^+$/FOLR1$^-$ cells (off-target), in %	Frequency of marker and negative reference, double positive cells in %
scFv-Fc 1	25.7	199.1	0,15	5,4	0,92
scFv-Fc 2	32.4	187.9	0,53	30,3	1,61
scFv-Fc 3	57.2	492.5	0,3	43,7	1,91
scFv-Fc 4	21.5	194.4	0,15	3,3	0,15
scFv-Fc 5	51.7	440.2	0,46	24,8	1,53
scFv-Fc 6	15.3	108.4	0,23	0,7	0,23
scFv-Fc 7	15.1	100.0	0,15	0,7	0,15
scFv-Fc 8	38.5	272.9	0,38	20,1	1,07
scFv-Fc 9	44.2	320.6	0,46	24	1,61
scFv-Fc 10	45.7	358.9	0,38	21,4	1,45
scFv-Fc 11	40.8	265.4	0,38	27,1	1,45
scFv-Fc 12	21.2	108.4	0,23	6,8	0,99
scFv-Fc 13	33.4	190.7	0,53	14,9	1,15
scFv-Fc 14	22.6	140.2	0,45	4,7	0,76
scFv-Fc 15	29.6	215.9	0,53	8,9	1,15
scFv-Fc 16	8.1	47.7	0	0,1	0
scFv-Fc 17	21.6	112.1	0,53	7,6	1,07
scFv-Fc 18	30.1	212.1	0,53	9	0,69
scFv-Fc 19	22.9	142.1	0,53	6,1	0,99
FOLR1 (positive reference)	14.6	100.0	N/A	N/A	0
VH36 (negative reference)	14.0	78.5	N/A	N/A	N/A

Supplementary Table S1C

Candidate	Mean intensity, in a.u.	Relative intensity to positive reference, in %	Frequency of scFv-Fc+/FOLR1+, double positive cells in %	Frequency of scFv-Fc+/FOLR1- cells (off-target), in %	Frequency of marker and negative reference, double positive cells in %
scFv-Fc 1	3119.0	73.3	100	0	0
scFv-Fc 2	4381.5	102.9	100	0	0
scFv-Fc 3	1913.8	45.0	100	0	0
scFv-Fc 4	1257.6	29.5	100	0	0
scFv-Fc 5	770.8	18.1	100	0	0
scFv-Fc 6	20.7	0.5	0	0	0
scFv-Fc 7	490.1	11.5	98	0	0
scFv-Fc 8	307.1	7.2	47	0	0
scFv-Fc 9	442.6	10.4	91	0	0
scFv-Fc 10	290.3	6.8	85	0	0
scFv-Fc 11	169.7	4.0	40	0	0
scFv-Fc 12	3520.0	82.7	100	0	0
scFv-Fc 13	97.3	2.3	1	0	0
scFv-Fc 14	488.6	11.5	98	0	0
scFv-Fc 15	83.5	2.0	1	0	0
scFv-Fc 16	8.7	0.2	0	0	0
scFv-Fc 17	428.1	10.1	100	0	0
scFv-Fc 18	88.3	2.1	0	0	0
scFv-Fc 19	608.4	14.3	100	0	0
FOLR1 (positive reference)	4257.3	100.0	N/A	N/A	0
VH36 (negative reference)	68.6	1.6	N/A	N/A	N/A

Supplementary Table S1D

Candidate	Mean intensity, in a.u.	Relative intensity to positive reference, in %	Frequency of scFv-Fc+/FOLR1+, double positive cells in %	Frequency of scFv-Fc+/FOLR1- cells (off-target), in %	Frequency of marker and negative reference, double positive cells in %
scFv-Fc 1	85.4	46.9	43,29	20.81	1,45
scFv-Fc 2	153.5	84.4	57,69	37.03	1,49
scFv-Fc 3	249.3	137.0	58,13	38.05	1,78
scFv-Fc 4	50.4	27.7	25,47	8.24	1,6
scFv-Fc 5	140.9	77.4	53,79	34.51	1,78
scFv-Fc 6	10.9	6.0	0,04	0.07	0
scFv-Fc 7	18.1	10.0	2,08	0.0	0,14
scFv-Fc 8	44.8	24.6	17,57	6.27	0,87
scFv-Fc 9	123.9	68.1	45,12	13.45	1,74
scFv-Fc 10	43.7	24.0	19,10	6.63	0
scFv-Fc 11	129.8	71.3	55,28	34.66	1,67
scFv-Fc 12	109.7	60.3	45,15	5.10	1,49
scFv-Fc 13	20.8	11.4	0,51	0.58	0,18
scFv-Fc 14	49.3	27.1	25,15	7.73	1,05
scFv-Fc 15	37.8	20.8	14,18	7.33	0,65
scFv-Fc 16	15.7	8.6	0,18	0.07	0,03
scFv-Fc 17	133.2	73.2	61,70	32.65	1,67
scFv-Fc 18	18.1	9.9	0,18	0.07	0,03
scFv-Fc 19	32.7	18.0	17,78	1.09	0,8
FOLR1 (positive reference)	181.9	100.0	N/A	N/A	1.42
VH36 (negative reference)	25.8	14.2	N/A	N/A	N/A

Supplementary Table S1E

Candidate	Mean intensity, in a.u.	Relative intensity to positive reference, in %	Frequency of scFv-Fc^{+}/FOLR1^{+}, double positive cells in %	Frequency of scFv-Fc^{+}/FOLR1^{-} cells (off-target), in %	Frequency of marker and negative reference, double positive cells in %
scFv-Fc 1	178.9	33.8	84,34	1.24	1,52
scFv-Fc 2	234.4	44.3	95,59	2.98	1,55
scFv-Fc 3	217.8	41.2	95,40	3.49	1,55
scFv-Fc 4	56.8	10.7	32,80	24.41	1,46
scFv-Fc 5	95.0	18.0	71,31	1.98	1,51
scFv-Fc 6	18.0	3.4	0,95	0.10	0,51
scFv-Fc 7	23.9	4.5	0,44	0.02	0,14
scFv-Fc 8	37.1	7.0	8,41	0.48	0,98
scFv-Fc 9	914.6	172.9	96,41	3.47	1,55
scFv-Fc 10	36.0	6.8	9,71	0.48	0,95
scFv-Fc 11	88.9	16.8	61,56	2.65	1,51
scFv-Fc 12	124.0	23.5	75,21	0.62	1,46
scFv-Fc 13	22.7	4.3	1,61	0.13	0,57
scFv-Fc 14	56.8	10.7	23,47	0.95	1,13
scFv-Fc 15	26.7	5.0	4,75	0.41	0,78
scFv-Fc 16	18.3	3.5	0,98	0.06	0,73
scFv-Fc 17	99.8	18.9	83,31	2.66	1,54
scFv-Fc 18	29.3	5.5	21,35	0.19	0,76
scFv-Fc 19	47.5	9.0	6,37	0.33	1,06
FOLR1 (positive reference)	528.9	100.0	N/A	N/A	1,52
VH36 (negative reference)	32.7	6.2	N/A	N/A	N/A

Supplementary Table S1F

Candidate	Mean intensity, in a.u.	Relative intensity to positive reference, in %	Frequency of scFv-Fc+/FOLR1+, double positive cells in %	Frequency of scFv-Fc+/FOLR1- cells (off-target), in %	Frequency of marker and negative reference, double positive cells in %
scFv-Fc 1	56.6	229.4	0	0.76	0,38
scFv-Fc 2	99.2	402.2	1	29.38	0,61
scFv-Fc 3	128.1	519.2	0	59.98	0,76
scFv-Fc 4	48.2	195.4	0	0.23	0,08
scFv-Fc 5	72.0	292.1	0	4.40	0,23
scFv-Fc 6	25.0	101.2	0	0.00	0,45
scFv-Fc 7	26.5	107.6	0	0.00	0,00
scFv-Fc 8	54.9	222.6	0	0.46	0,83
scFv-Fc 9	121.5	492.4	0	21.87	0,38
scFv-Fc 10	56.1	227.5	0	0.68	0,08
scFv-Fc 11	85.9	348.2	0	12.07	0,38
scFv-Fc 12	46.0	186.5	0	0.30	0,00
scFv-Fc 13	43.0	174.2	0	0.30	0,00
scFv-Fc 14	41.1	166.7	0	0.23	0,00
scFv-Fc 15	22.8	92.5	0	0.15	0,00
scFv-Fc 16	20.3	82.2	0	0.00	0,08
scFv-Fc 17	69.7	282.4	0.15	6.82	0,15
scFv-Fc 18	30.7	124.4	0	0.08	0,00
scFv-Fc 19	28.8	116.7	0	0.00	0,00
FOLR1 (positive reference)	24.7	100.0	N/A	N/A	0,00
VH36 (negative reference)	59.3	240.5	N/A	N/A	N/A

Supplementary Table S2. Summary of the identification and selection workflow assays for novel, fully human CAR T cell lead candidates. Complementary approaches and assays were employed to generate specific binders and efficient as well as safe CAR T cells. Here the different assay results of the positive controls and the 20 binder candidates selected after flow cytometric analysis are summarized. *Killing (WT): green: reduction of fluorescence intensity (end vs. start of round) (3 donors), yellow: reduction of fluorescence intensity (2 donors), red: reduction of fluorescence intensity (≤1 donor). **Absence of killing (KO): green: increase of fluorescence intensity (end vs. start of round) (0 donors), red: increase of fluorescence intensity (≥1 donor). ***IFN-γ, round 1: green >400 pg/ml (3 donors), yellow: >400 pg/ml (2 donors), red: <400 pg/ml (2-3 donors). ****Expansion: green: >50,000 LNGFR+ T cells (3 donors), yellow: >50,000 LNGFR+ T cells (2 donors), red: >50,000 LNGFR+ T cells (≤1 donor). *****IFN-γ, round 3: green >2,500 pg/ml (3 donors), yellow: >2,500 pg/ml (2 donors), red: <2,500 pg/ml (2-3 donors). #Killing (WT): green: increased reduction of GAC to positive crtl, yellow: comparable reduction of GAC to positive control, red: decreased reduction of GAC to positive control. ##Absence of killing (KO): green: pass, red: fail; yellow: intermediate; grey: non-applicable; ✝cross reactivity: on-target (red < 50% ≤ green); off-target (green ≤ 10% < red)

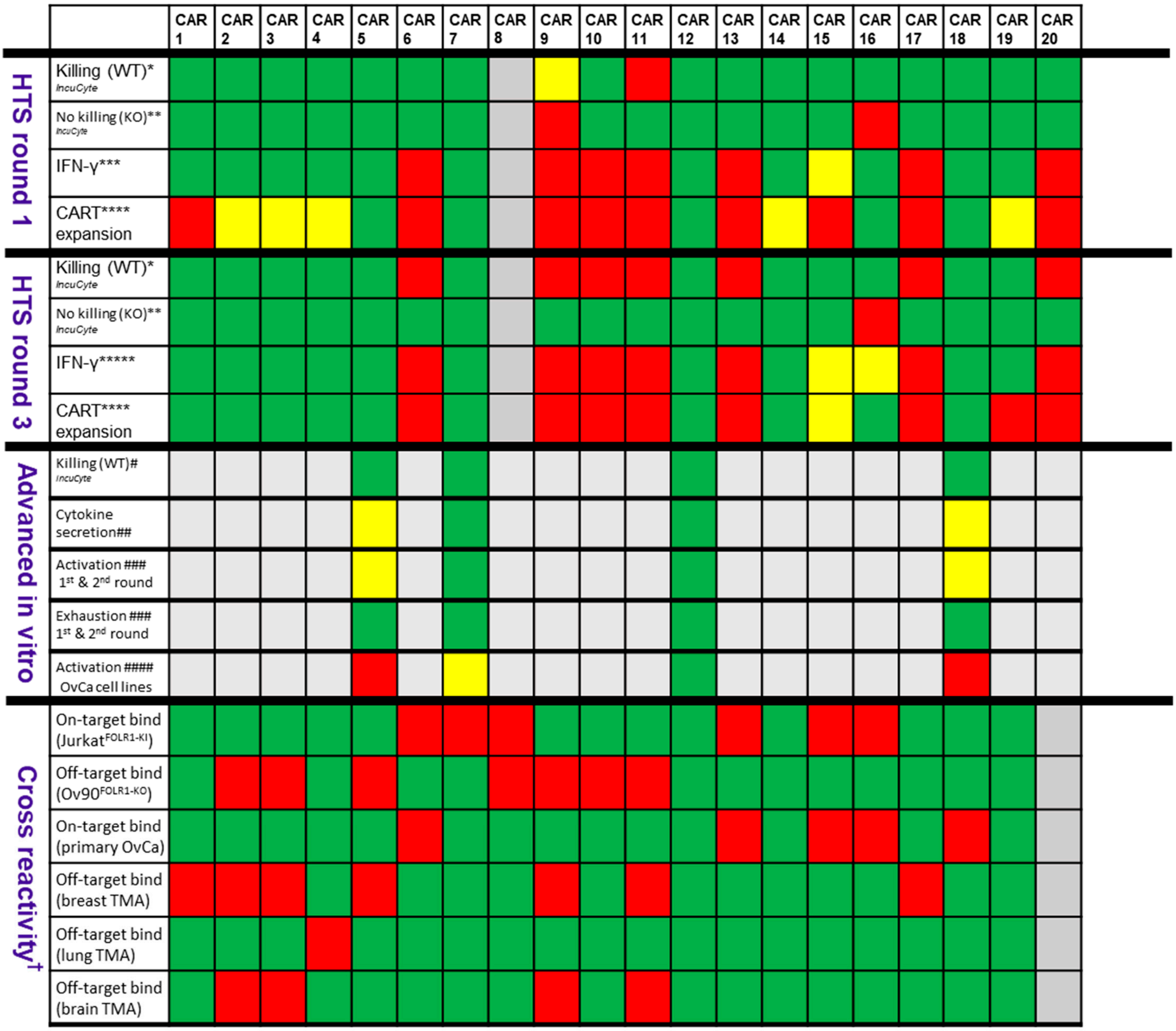
CAR 1
CAR 2
CAR 3
CAR 4
CAR 5
CAR 6
CAR 7
CAR 8
CAR 9
CAR 10
CAR 11
CAR 12
CAR 13
CAR 14
CAR 15
CAR 16
CAR 17
CAR 18
CAR 19
CAR 20
HTS round 1
Killing (WT)* IncuCyte
No killing (KO)** IncuCyte
IFN-γ***
CART**** expansion
HTS round 3
Killing (WT)* IncuCyte
No killing (KO)** IncuCyte
IFN-γ*****
CART**** expansion
Advanced in vitro
Killing (WT)# IncuCyte
Cytokine secretion##
Activation ### 1st & 2nd round
Exhaustion ### 1st & 2nd round
Activation #### OvCa cell lines
Cross reactivity†
On-target bind (Jurkat FOLR1-KI)
Off-target bind (Ov90 FOLR1-KO)
On-target bind (primary OvCa)
Off-target bind (breast TMA)
Off-target bind (lung TMA)
Off-target bind (brain TMA)

Supplementary Table S3. Reagent list

Material	Ordering number	Product Name	Provider
24 deep well plates	WHA770 15110	Whatman® UNIPLATE microplates	Sigma-Aldrich/Merck
			(Taufkirchen, Germany)
384 ELISA Platten	781094	Greiner microplate 384 well, flat µClear® bottom, high binding, white	Greiner BioOne
			(Kremsmuenster, Austria)
4-1BB APC	130-110-764	CD137 Antibody, anti-human, REAfinity™, REA765	Miltenyi Biotec (Bergisch Gladbach, Germany)
			(Bergisch Gladbach, Germany)
7-AAD	130-111-568	7-AAD Staining Solution	Miltenyi Biotec (Bergisch Gladbach, Germany)
			(Bergisch Gladbach, Germany)
96 well Nunc MaxiSorb ELISA plates	735-0034	MicroWell™ 96-Well-Platten, Nunc-Immuno™	VWR
			(Darmstadt, Germany)
96 well plates PP	655201	MICROPLATE, 96 WELL, PP, F-BOTTOM	Greiner BioOne (Kremsmuenster, Austria)
96-well Culture Microplate	353072	Falcon® 96-well Clear Flat Bottom TC-treated Culture Microplate	Corning Falcon
			(Duderstadt, Germany)
96-well Plates, skirted	9283661	TWIN.TEC PCR PLATE 96 SKIRTED	TH Geyer
			(Hoxter, Germany)
96-well Plates, unskirted	7671290	TWIN.TEC 96WELL PCR-PLATTEN colourless, UNSKIRTED, LOW-PROFILE	TH Geyer
			(Hoxter, Germany)
AffinitPure $F(ab')_2$ Fragment IgG-PE	109-116-097	R-Phycoerythrin AffiniPure™ $F(ab')_2$ Fragment Goat Anti-Human IgG, $F(ab')_2$ fragment specific	Jackson ImmunoResearch
			(Cambridgeshire, UK)
Agar	A5054	Select Agar	Sigma-Aldrich/Merck
			(Taufkirchen, Germany)
Agarose	9920	AGAROSE STANDARD, FOR ELECTROPHORESIS	TH Geyer
			(Hoxter, Germany)
Aluminium foil	900 310	Aluminium sealing foil	HJ-Bioanalytik
			(Erkelenz Germany)
amicon tube 30 kDa	UFC9030	Amicon® Ultra Centrifugal Filter, 30 kDa MWCO	Sigma-Aldrich/Merck
			(Taufkirchen, Germany)

Ampicillin	A0839	Ampicillin Sodium Salt *BioChemica*	AppliChem
			(Darmstadt, Germany)
anti c-Myc-HRP antibody	130-092-113	c-myc Antibody, HRP	Miltenyi Biotec (Bergisch Gladbach, Germany)
Binding Buffer		High salt buffer: 1.5 M Glycine, 3 M NaCl, pH8.9	
Biotin PE	130-111-068	Biotin Antibody, REAfinity™ (REA746)	Miltenyi Biotec (Bergisch Gladbach, Germany)
Bluo-Gal	50375,1	Bluo-Gal, pure	Biomol
			(Hamburg, Germany)
BSA	130-091-376	MACS® BSA Stock Solution	Miltenyi Biotec (Bergisch Gladbach, Germany)
CD137 Vio Bright FITC	130-110-765	CD137 Antibody, anti-human, REAfinity™ (REA765)	Miltenyi Biotec (Bergisch Gladbach, Germany)
CD223 Vio Bright B515	130-120-012	CD223 Antibody, anti-human, REAfinity™ (REA351)	Miltenyi Biotec (Bergisch Gladbach, Germany)
CD223 VioBlue	130-118-549	CD223 Antibody, anti-human, REAfinity™ (REA351)	Miltenyi Biotec (Bergisch Gladbach, Germany)
CD25 PE-Vio 770	130-114-541	CD25 Antibody, anti-human, REAfinity™ (REA570)	Miltenyi Biotec (Bergisch Gladbach, Germany)
CD25 PE-Vio 770	130-116-205	CD25 Antibody, anti-human, REAfinity™ (REA945)	Miltenyi Biotec (Bergisch Gladbach, Germany)
CD271 (LNGFR) PE	130-112-601	CD271 (LNGFR) Antibody, anti-human, REAfinity™ (REA844)	Miltenyi Biotec (Bergisch Gladbach, Germany)
CD3 VioGreen	130-113-142	CD3 Antibody, anti-human, REAfinity™ (REA613)	Miltenyi Biotec (Bergisch Gladbach, Germany)
CD326 PE	130-110-999	CD326 (EpCAM) Antibody, anti-human, REAfinity™ (REA764)	Miltenyi Biotec (Bergisch Gladbach, Germany)
CD4 VioGreen	130-113-230	CD4 Antibody, anti-human, REAfinity™ (REA623)	Miltenyi Biotec (Bergisch Gladbach, Germany)
CD4+ TC Iso	130-096-533	CD4+ T Cell Isolation Kit, human	Miltenyi Biotec (Bergisch Gladbach, Germany)
CD45 PE	130-110-632	CD45 Antibody, anti-human, REAfinity™ (REA747)	Miltenyi Biotec (Bergisch Gladbach, Germany)
CD56 APC	130-113-310	CD56 Antibody, anti-human, REAfinity™ (REA196)	Miltenyi Biotec (Bergisch Gladbach, Germany)
CD69 VioBlue	130-112-610	CD69 Antibody, anti-human, REAfinity™ (REA824)	Miltenyi Biotec (Bergisch Gladbach, Germany)
CD8 APC-Vio 770	130-110-681	CD8 Antibody, anti-human, REAfinity™ (REA734)	Miltenyi Biotec (Bergisch Gladbach, Germany)
CD90 APC	130-114-861	CD90 Antibody, anti-human, REAfinity™ (REA897)	Miltenyi Biotec (Bergisch Gladbach, Germany)
CD90 PE	130-114-860	CD90 Antibody, anti-human, REAfinity™ (REA897)	Miltenyi Biotec (Bergisch Gladbach, Germany)
Cell Trace™ FarRed	C34572	CellTrace™ Far Red Cell Proliferation Kit, for flow cytometry	Thermo Fisher Scientific
			(Waltham, MA, USA)
CellTrace™ Violet	C34557	CellTrace™ Violet Cell Proliferation Kit, for flow cytometry	Thermo Fisher Scientific
			(Waltham, MA, USA)

COPYcheck kit	130-128-157	MACS® COPYcheck Kit, human	Miltenyi Biotec (Bergisch Gladbach, Germany)
DAPI	130-111-570	DAPI Staining Solution	Miltenyi Biotec (Bergisch Gladbach, Germany)
Deep well plates, 96 square well	732-3325	Deep Well Plates, Square Well	VWR
			(Darmstadt, Germany)
Deepwell Plates	130-126-865	MACSwell™ Deepwell Plates	Miltenyi Biotec (Bergisch Gladbach, Germany)
DMSO	D2650	Dimethylsulfoxid Hybri-Max™, sterile-filtered, BioReagent, suitable for hybridoma, ≥99.7%	Sigma-Aldrich/Merck
			(Taufkirchen, Germany)
Elution Buffer	J67349-AK	Glycine chloric acid, 0.1 M buffer solution, pH 3.0	Thermo Fisher Scientific
			(Waltham, MA, USA)
Ethidium bromide	HP47.1	Ethidium bromide solution 0.025 % in dropper bottle	Carl Roth
			(Karlsruhe, Germany)
EXCIMUS Standardicus	BS-2020-5002	EXIMUS Standardicus FBS solution, sterile filtered	Catus Biotech GmbH
			(Tutzingen, Germany)
Expi293 expression system kit	A14635	Expi293 expression system kit	Thermo Fisher Scientific
			(Waltham, MA, USA)
FarletuzumAb (FR1 mAb)	PX-TA1207	Farletuzumab Biosimilar - Anti-FOLR1 mAb - Research Grade	ProteoGenix SAS
			(Newark, DE, USA)
FcR Blocking	130-059-901	FcR Blocking Reagent, human	Miltenyi Biotec (Bergisch Gladbach, Germany)
FOLR1	908304	PE anti-FOLR1 Antibody (LK26)	Biolegend
			(San Diego, CA, USA)
Glucose	G7021	D-(+)-Glucose	Sigma-Aldrich/Merck
Glycerin	G5516	Glycerin for molecular biology, ≥99.0%	(Taufkirchen, Germany)
H2SO4	0971.1	Schwefelsäure 96%	Carl Roth
			(Karlsruhe, Germany)
hFOLR1	F01-H5253	Human FOLR1 Protein, Fc Tag (MALS verified)	Acro Biosystems
			(Basel, Switzerland)
hFOLR2	FO2-H5223	Human FOLR2 Protein, His Tag	Acro Biosystems
			(Basel, Switzerland)
hFOLR3	5319-FR-050	Recombinant Human FOLR3 Protein	TH Geyer
			(Hoxter, Germany)
hIgG1 Fc	FCC-H5214	Human IgG1 Fc Protein, Tag Free (MALS verified)	Amsbio
			(Massagno, Switzerland)
IL-15	130-095-760	Human IL-15, research grade	Miltenyi Biotec (Bergisch Gladbach, Germany)

IL-15 (GMP, 1000x)	170-076-114	MACS® GMP Recombinant Human IL-15	Miltenyi Biotec (Bergisch Gladbach, Germany)
IL-7	130-095-367	Human IL-7, research grade	Miltenyi Biotec (Bergisch Gladbach, Germany)
IL-7 (GMP, 1000x)	170-076-111	MACS® GMP Recombinant Human IL-7	Miltenyi Biotec (Bergisch Gladbach, Germany)
IPTG	I6758	IPTG ≥99% (TLC), ≤0.1% Dioxane	Sigma-Aldrich/Merck
			(Taufkirchen, Germany)
Kanamycin	T832.5	Kanamycin sulphate	Carl Roth
			(Karlsruhe, Germany)
L-Glutamine	BE17-605E	Lonza L-Glutamine, 200 mM	Lonza
			(Basel, Switzerland)
Ligase	M0202L	T4 DNA Ligase	NEB
			(Ipswich, MA, USA)
M13K07 Helperphage	PH010L-ADL	M13KO7 Helper Phage, purified (2 x10^{12} pfu/ml)	BioCat
			(Heidelberg, Germany)
MACSima Running Buffer	130-121-565	MACSima™ Running Buffer	Miltenyi Biotec (Bergisch Gladbach, Germany)
MACSima Storage Solution	130-092-748	MACSQuant®/MACSima™ Storage Solution	Miltenyi Biotec (Bergisch Gladbach, Germany)
MACSima System Buffer	130-125-315	MACSima™ System Buffer	Miltenyi Biotec (Bergisch Gladbach, Germany)
MACSPlex 12 kit	130-099-169	MACSPlex Cytokine 12 Kit, human	Miltenyi Biotec (Bergisch Gladbach, Germany)
MACSwell 1	130-124-673	MACSwell™ One Imaging Frames	Miltenyi Biotec (Bergisch Gladbach, Germany)
MACSwell 24	130-124-677	MACSwell™ 24 Imaging Plates	Miltenyi Biotec (Bergisch Gladbach, Germany)
MACSwell 4	130-124-676	MACSwell™ Four Imaging Frames	Miltenyi Biotec (Bergisch Gladbach, Germany)
MACSwell Sealing Foils	130-126-866	MACSwell™ Sealing Foils	Miltenyi Biotec (Bergisch Gladbach, Germany)
MCDB 105 Medium	117-500	MCDB 105 Medium, Unmodified	Cell Applications, Inc.
			(San Diego, CA, USA)
MES	M1511	Sodium 2-mercaptoethanesulfonate, analytical standard, ≥98.0% (titration)	Sigma-Aldrich/Merck
			(Taufkirchen, Germany)
mFOLR1	FO1-M5225	Mouse FOLR1 Protein, His Tag	Acro Biosystems
			(Basel, Switzerland)
MQ calibration beads	130-093-607	MACSQuant® Calibration Beads	Miltenyi Biotec (Bergisch Gladbach, Germany)
Multi-96 Columns	130-092-445	Multi-96 Columns, molecular (4×96)	Miltenyi Biotec (Bergisch Gladbach, Germany)
NaCl	9265.2	Natriumchlorid ≥99,8 %	Carl Roth
			(Karlsruhe, Germany)

Naive Pan TC Iso	130-097-095	Naive Pan T Cell Isolation Kit, human	Miltenyi Biotec (Bergisch Gladbach, Germany)
NAP10	10228284	Cytiva NAP™ Columns, NAP-10	Thermo Fisher Scientific
			(Waltham, MA, USA)
NcoI-HF	R3193L	NcoI High-Fidelity (HF®) restriction enzymes	NEB
			(Ipswich, MA, USA)
NEB Stable	C3040H	NEB® Stable Competent E. coli (High Efficiency)	NEB
			(Ipswich, MA, USA)
NEB 10-beta/Stable Outgrowth Medium	B9035S	NEB 10-beta/Stable Outgrowth Medium	NEB
			(Ipswich, MA, USA)
Neutralization Buffer	J62085-K2	TRIS, 1.0 M buffer solution, pH 9.0	Thermo Fisher Scientific
			(Waltham, MA, USA)
Non-Woven Foil	900 390	Nonwoven foil	HJ-Bioanalytik
			(Erkelenz Germany)
NotI-HF	R3189L	NotI High-Fidelity (HF®) restriction enzymes	NEB
			(Ipswich, MA, USA)
Nuclease free Water	B1500L	Nuclease-free Water	NEB
			(Ipswich, MA, USA)
NucleoSpin® Gel and PCR Clean-up	740609	NucleoSpin Gel and PCR Clean-up, Mini kit for gel extraction or PCR clean up	Macherey-Nagel
			(Duren, Germany)
Pan TC Iso	130-096-535	Pan T Cell Isolation Kit, human	Miltenyi Biotec (Bergisch Gladbach, Germany)
Pancoll	P04-601000	Pancoll human, Density: 1.077 g/ml, sterile filtered	PAN Biotech
			(Aidenbach, Germany)
PBS	14190144	DPBS, ohne Kalzium, ohne Magnesium	Thermo Fisher Scientific
			(Waltham, MA, USA)
PBS/EDTA Buffer	200-070-029	CliniMACS® PBS/EDTA Buffer (2x3L)	Miltenyi Biotec (Bergisch Gladbach, Germany)
PD1 PE-Vio 770	130-117-698	CD279 (PD1) Antibody, anti-human, PD1.3.1.3	Miltenyi Biotec (Bergisch Gladbach, Germany)
PE	ABD-2558	PE [R-Phycoerythrin]	AAT Bioquest
			(Sunnyvale, CA, USA)
Pen/Strep	15140122	Penicillin-Streptomycin (10.000 U/ml)	Thermo Fisher Scientific
			(Waltham, MA, USA)
pFUSE-hIgG1-Fc2	pfuse-hg1fc2	pFUSE-hIgG1-Fc2 plasmid designed for the construction of Fc-Fusion proteins	Invivogen
			(San Diego, CA, USA)
Phusion	F-530XL	Phusion™ High-Fidelity DNA Polymerase (2 U/μL)	Thermo Fisher Scientific
			(Waltham, MA, USA)

Poloxamer 188	P5556-100ML	Poloxamer 188 solution	Sigma-Aldrich/Merck
			(Taufkirchen, Germany)
Protein A Microbeads	130-071-001	µMACS™ Protein A MicroBeads	Miltenyi Biotec (Bergisch Gladbach, Germany)
Protein A PhyTip® columns	NC2129541	Protein A PhyTip® columns	Biotage
			(Uppsala, Sweden)
Protein G Microbeads	130-071-101	µMACS™ Protein G MicroBeads	Miltenyi Biotec (Bergisch Gladbach, Germany)
QIAGEN Plasmid Plus 96 Miniprep Kit	16181	QIAGEN Plasmid Plus 96 Miniprep Kit	Qiagen
			(Hilden, Germany
Reduction/Conjugation Buffer		100 mM Na_2HPO_4 pH6.8, 50 mM NaCl, 1 mM EDTA	
RPMI 1640	L0501-500	Cell culture media, RPMI 1640	Biowest
			(Bradenton, FL, USA)
rSAP	M0371L	Shrimp Alkaline Phosphatase (rSAP)	NEB
			(Ipswich, MA, USA)
SMCC	M5525	4-(N-Maleimidomethyl)cyclohexan-Carboxylsäure N-hydroxysuccinimid-Ester, ≥98%, powder	Sigma-Aldrich/Merck
			(Taufkirchen, Germany)
TCEP	T2556	Tris-(2-Carboxyethyl)phosphine, Hydrochloride (TCEP)	Thermo Fisher Scientific
			(Waltham, MA, USA)
TexMACS Medium	130-097-196	TexMACS ™ Medium, (research grade)	Miltenyi Biotec (Bergisch Gladbach, Germany)
TG1 Bacteria	60502-1-LU	Phage Display optimized TG1 Electrocompetent Cells	Lucigen
			(Middelton, WI, USA)
Tim3 APC	130-119-781	CD366 (TIM-3) Antibody, anti-human, REAfinity™ (REA635)	Miltenyi Biotec (Bergisch Gladbach, Germany)
Tim3 PE	130-117-364	CD366 (TIM-3) Antibody, anti-human, REAfinity™ (REA635)	Miltenyi Biotec (Bergisch Gladbach, Germany)
TMB Substrate	34029	1-Step™ Ultra TMB	Thermo Fisher Scientific
			(Waltham, MA, USA)
TransAct	130-111-160	T Cell TransAct™, human	Miltenyi Biotec (Bergisch Gladbach, Germany)
Triton X-100	X100	Triton™ X-100	Sigma-Aldrich/Merck
Trypsin	T8802	Trypsin from bovine pancreas	(Taufkirchen, Germany)
Trypsin-EDTA	T4049-100ML	Trypsin-EDTA Solution	Sigma-Aldrich/Merck
Tryptone / Pepton	71280-3	Veggie Peptone - Novagen	Merck
			(Darmstadt, Germany)
Tween 20	9127,1	Tween® 20, 250 g	Carl Roth
X-Gal	B4252-250MG	5-Brom-4-Chlor-3-indolyl-β-D-Galactopyranosid	Sigma-Aldrich/Merck

Yeast extract	71279	Veggie Yeast Extract - Novagen	(Taufkirchen, Germany)
Zeocin	ant-zn-1	Zeocin® (powder)	InvivoGen
			(San Diego, CA, USA)
ZR-96 Zymoclean Gel DNA Recovery Kit	D4022	ZR-96 Zymoclean Gel DNA Recovery Kit	Zymo (Freiburg, Germany)
